# Extreme Extensibility in Physically Cross-Linked Nanocomposite Hydrogels Leveraging Dynamic Polymer–Nanoparticle Interactions

**DOI:** 10.1021/acs.macromol.2c00649

**Published:** 2022-08-16

**Authors:** Abigail K. Grosskopf, Joseph L. Mann, Julie Baillet, Hector Lopez Hernandez, Anton A. A. Autzen, Anthony C. Yu, Eric A. Appel

**Affiliations:** † Department of Chemical Engineering, Stanford University, Stanford, California 94305, United States; ‡ Department of Materials Science and Engineering, Stanford University, Stanford, California 94305, United States; § CNRS, Bordeaux INP, LCPO, University of Bordeaux, Pessac 33600, France; ∥ Department of Health Technology, Technical University of Denmark, 2800 Lyngby, Denmark; ⊥ Department of Bioengineering, Stanford University, Stanford, California 94305, United States; # Department of Pediatrics- Endocrinology, Stanford University, Stanford, California 94305, United States; % ChEM-H Institute, Stanford University, Stanford, California 94305, United States; & Woods Institute for the Environment, Stanford University, Stanford, California 94305, United States

## Abstract

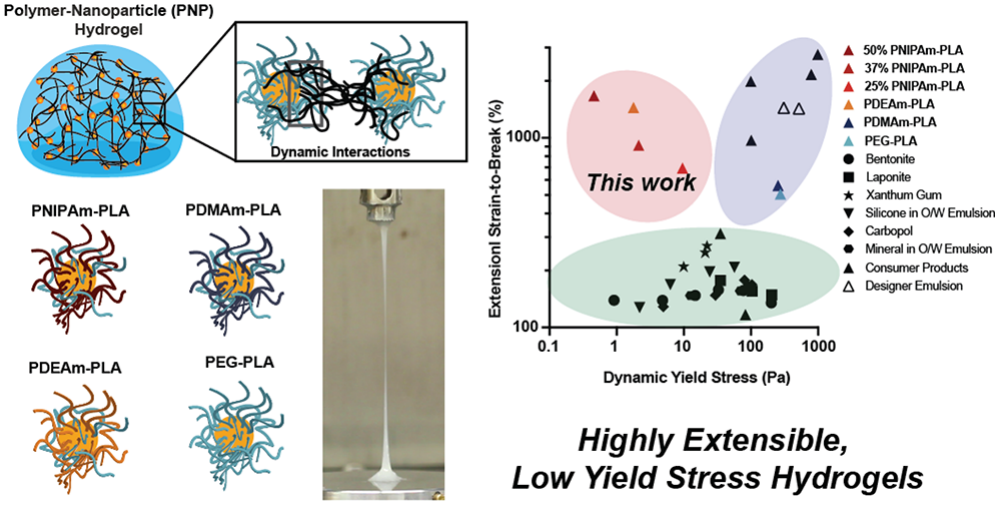

Designing yield stress fluids to exhibit desired functional properties is an integral challenge in many applications such as 3D printing, drilling, food formulation, fiber spinning, adhesives, and injectable biomaterials. Extensibility in particular has been found to be a highly beneficial characteristic for materials in these applications; however, few highly extensible, high water content materials have been reported to date. Herein we engineer a class of high water content nanocomposite hydrogel materials leveraging multivalent, noncovalent, polymer–nanoparticle (PNP) interactions between modified cellulose polymers and biodegradable nanoparticles. We show that modulation of the chemical composition of the PNP hydrogels controls the dynamic cross-linking interactions within the polymer network and directly impacts yielding and viscoelastic responses. These materials can be engineered to stretch up to 2000% strain and occupy an unprecedented property regime for extensible yield stress fluids. Moreover, a dimensional analysis of the relationships between extensibility and the relaxation and recovery time scales of these nanocomposite hydrogels uncovers generalizable design criteria that will be critical for future development of extensible materials.

## Introduction

Designing yield stress fluids is crucial in many applications such as drilling fluids, 3D printing materials, food materials, fiber formulation, adhesives, and injectable biomaterials.^1−7^ These applications require materials with tailored viscoelastic and yielding properties, and it has been shown that multiple distinct relaxation behaviors and yield strength directly impact material performance. Indeed, material properties such as extreme compressibility, toughness, adhesivity, and extensibility can be achieved and tuned through careful understanding of the chemical interactions and dynamics that make up a material’s microstructure.^2,8−10^

In particular, the extensibility of yield stress fluids has been found to play a key role in various applications such as improving filament fidelity during 3D printing,^2^ reducing cell death or encapsulated protein damage during injection through needles,^11−13^ improving stable fiber formation during fiber spinning,^14^ and contributing to the function of adhesives.^6^ Yet, few yield stress fluids exhibit high extensibility while maintaining high water content—two properties often required for biological applications and mimicry of biological tissues. In particular, yield stress fluids with less pronounced yield stress behavior (<50 Pa) and measured high extensibility have yet to be reported to our knowledge.^15^ A high yield stress (>50 Pa) may hinder use in some applications like 3D printing or injectable gels where the initiation of flow must be facile to allow extrusion,^7^ but the presence of some yield stress is needed to maintain structure.^3,16^ Mucin or other biopolymer networks are the most likely natural candidates to fit these criteria,^17,18^ but these materials could not be made reproducibly in large batches for applications, so a synthetic and scalable alternative is needed. Additionally, the ability to engineer and control extensibility would enable optimization for specific applications.

In this work, we modulate the chemical composition of a class of hydrogels leveraging multivalent, noncovalent, polymer–nanoparticle (PNP) interactions between modified cellulose polymers and biodegradable to generate yield stress fluids exhibiting extreme extensibility. We use controlled radical polymerization and organocatalytic ring-opening polymerization techniques to synthesize four different copolymers and employ nanoprecipitation approaches to form biodegradable nanoparticles. We then prepare a series of PNP hydrogels from these building blocks that possess both high water content and tunable mechanical properties. We find that by modulating the PNP interactions at the nanoscale, the macrostructural properties can be precisely controlled to generate PNP hydrogel nanocomposite materials with unprecedented combinations of viscoelasticity, yielding, and extensibility. We identify key relationships between measurable rheological quantities and relevant time scales, providing insight critical design criteria for highly nonideal yet highly useful supramolecular materials systems through analysis of time scales.

## Results and Discussion

### Fabrication of Polymer–Nanoparticle Hydrogels

**Figure 1:**
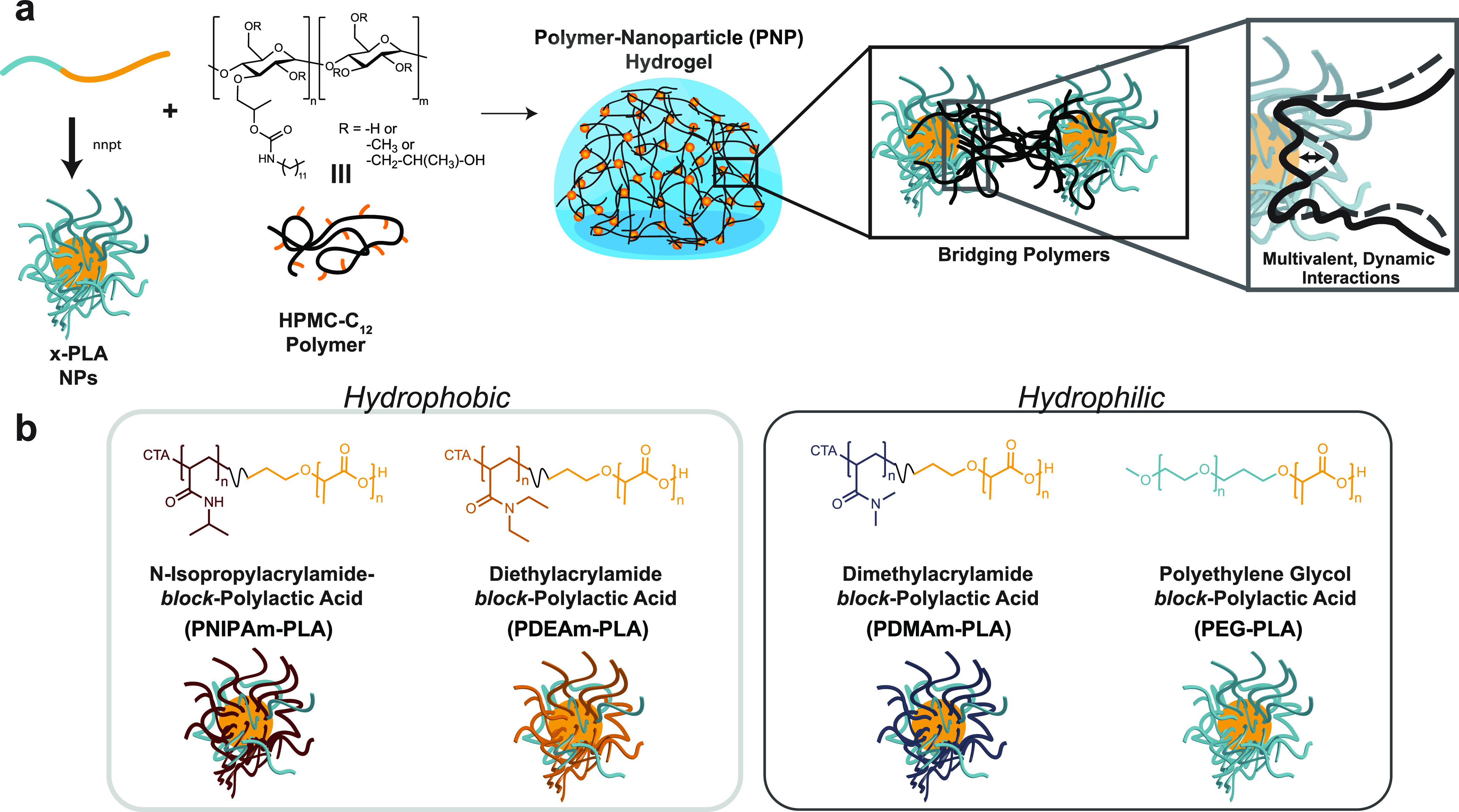
(a) Schematic illustrating polymer–nanoparticle (PNP) hydrogel formation upon mixing of nanoparticles and dodecyl-modified hydroxypropylmethylcellulose (HPMC-C_12_). Nanoparticles are formed by nanoprecipitation (nnpt) of diblock copolymers comprising a poly(lactic acid) (PLA) hydrophobic block and various water-soluble polymer blocks. (b) Chemical composition of diblock copolymers comprising multiple hydrophobic and hydrophilic water-soluble blocks investigated in this study for the preparation of PNP hydrogels.

PNP hydrogels are fabricated by mixing a solution of dodecyl-modified hydroxypropylmethylcellulose (HPMC-C_12_) with a solution of biodegradable nanoparticles comprising poly(ethylene glycol)-*b*-poly(lactic acid) (PEG–PLA NPs) (Figure 1).^19^ Upon mixing, dynamic multivalent interactions between the HPMC-C_12_ polymers and the PEG–PLA NPs generate physical cross-linking that yields robust hydrogels (Figure 1a). While PEG–PLA NPs have been used previously for many biological applications to deliver encapsulated cargo,^20,21^ in this system they serve as a structural building block to form the physically cross-linked hydrogel network. The self-assembled, entropy-driven cross-linking interactions within these materials yields temperature-invariant mechanical properties,^22^ and their dynamic structure enables facile injection through a needle or catheter.^7^ PNP hydrogels comprising these biodegradable PEG–PLA NPs have been highly useful for various biomedical applications ranging from controlled vaccine delivery, adhesion barriers to prevent postoperative scarring, and scaffolds for controlled cell delivery.^19,23−26^ While highly useful, these materials exhibit non-ideal behavior that cannot be fully captured with typical mechanical models; furthermore, the entropy-driven PNP-based cross-linking interactions in these materials make it impossible to characterize them by using standard time–temperature superposition approaches that are suitable for other physically cross-linked materials.^8,22,27,28^

In this study, we aimed to design a series of nanoparticles with different polymer coronas to investigate how modulation of the resulting PNP interactions impacts the mechanical properties of the resulting PNP hydrogels. In particular, we sought to assess whether changing the interaction dynamics could yield diverse mechanical properties and enhance extensibility of these materials. To explore this design space, we synthesized a series of diblock copolymers: (i) poly(*N*-isopropylacrylamide)-*b*-poly(lactic acid) (PNIPAm–PLA), (ii) poly(diethylacrylamide)-*b*-poly(lactic acid) (PDEAm–PLA), (iii) poly(dimethylacrylamide)-*b*-poly(lactic acid) (PDMAm–PLA), and (iv) PEG–PLA (Figure 1b). We chose to focus on polyacrylamide-based polymers on account of the broad array of nonionic and water-soluble monomers that are commercially available and exhibit tunable degrees of hydrophobicity.^29^ We selected the four diblock copolymers used in this study for several key physicochemical differences. First, PNIPAm and PDEAm are relatively hydrophobic polymers with log *P* ∼ 1, evidenced by their observable lower critical solution behavior at modest temperatures, while PDMAm and PEG are more hydrophilic with log *P* ∼ 0 (log *P* values estimated from PubChem). Chi parameters, if available, would also demonstrate differences in hydrophobicity for these polymers. Second, while all four polymers are capable of accepting hydrogen bonds, only PNIPAm is capable of hydrogen bond donation.

**Figure 2:**
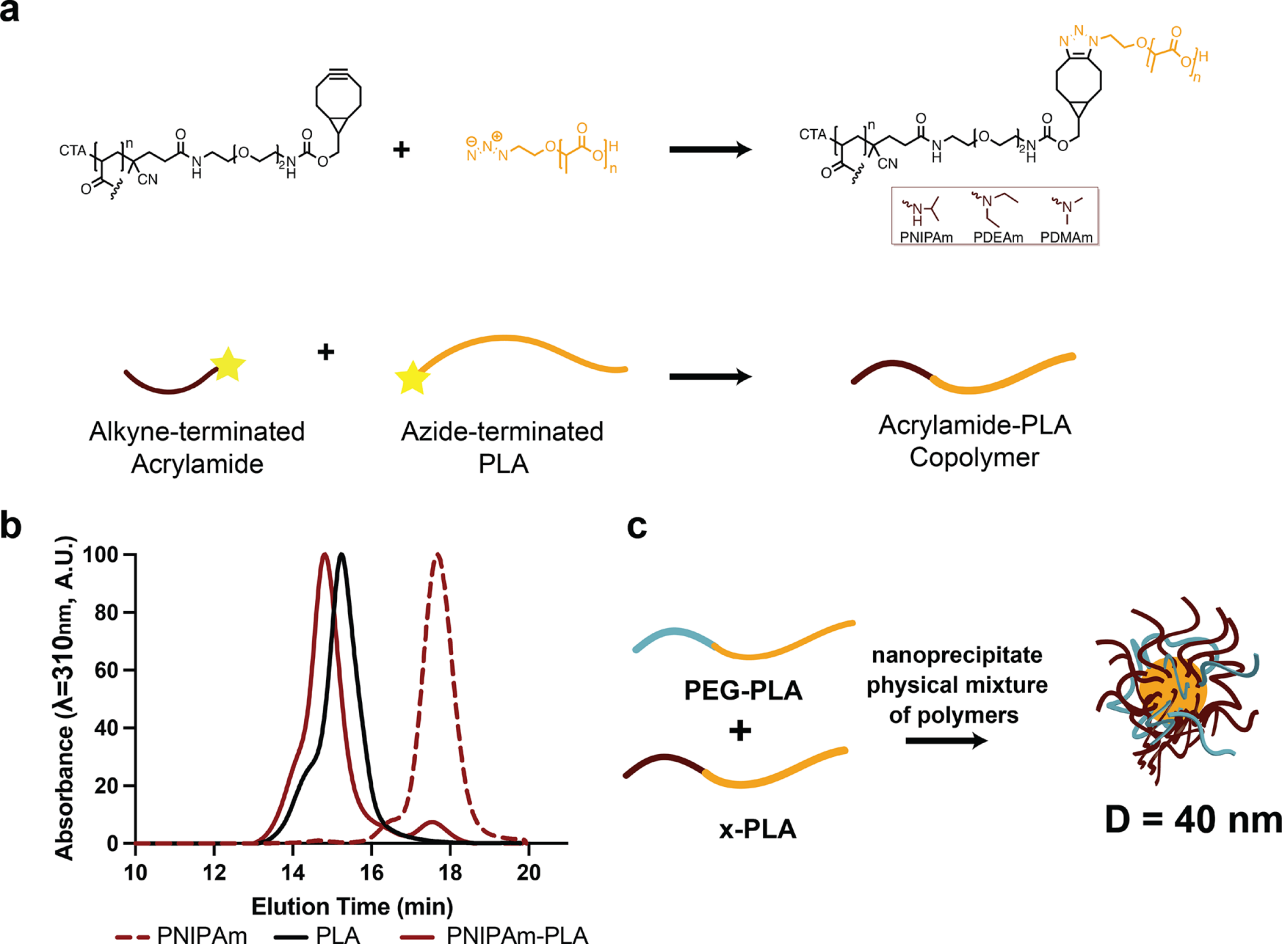
(a) Synthesis of copolymers through click conjugation of alkyne-terminated acrylamides and azide-terminated poly(lactic acid). (b) Size exclusion chromatography trace of PNIPAm, PLA, and the resulting copolymer, PNIPAm–PLA. (c) Schematic describing the formation of nanoparticles composed of a physical mixture of acrylamide-based copolymers and PEG–PLA.

Polyacrylamide-based copolymers with relatively low molecular weight PLA blocks (<5 kDa) have been synthesized previously with tin catalysts that are often unsuitable for biological applications due to toxicity concerns and challenging removal of the catalyst postsynthesis.^30−33^ Here we aimed to create diblock copolymers with relatively high molecular weight PLA blocks (20 kDa) to support preparation of highly stable nanoparticles using more biocompatible synthetic methods. To synthesize polyacrylamide-based copolymers, we leveraged copper-free click chemistry and fractional precipitation techniques (Figures 2a, S1, and S2).^34−36^ Polyacrylamide derivatives were first synthesized by using reversible addition–fragmentation chain transfer (RAFT) polymerization techniques^37^ affording controlled molecular weights and low dispersity. A bicyclononyne derivative (BCN-amine) was then conjugated to the end-group of the polymers. PLA was polymerized from azidoethanol by using an organocatalytic ring-opening polymerization technique with 1,8-diazabicycloundec-7-ene (DBU) as a catalyst.^38^ These two polymers were then coupled together to form diblock copolymers by using copper-free strain-promoted azide–alkyne cycloaddition (SPAAC).^35,39^ Fractional precipitation in solvents of varying polarity were then performed to isolate the desired product from residual homopolymer to yield monodisperse diblock copolymers (**D < 1.12) with a water-soluble block of 5–6 kDa and a PLA block of 17–20 kDa (Figure 2b, Table S1, and Figure S3).

Nanoprecipitation of these copolymers was then performed to generate NPs with various water-soluble coronas. While PDMAm–PLA was able to form stable NPs by using this approach, neither of the more hydrophobic block copolymers PNIPAm–PLA and PDEAm–PLA were able to form stable NPs, and immediate aggregation was observed upon concentration. To generate stable NPs, a 1:1 (w:w) physical mixture of these polyacrylamide-based copolymers with PEG–PLA was used (Figure 2c and Table S2). For each of these materials, monodisperse NPs exhibiting a hydrodynamic diameter of ∼40 nm (PDI < 0.1) were prepared (Tables S3–S7). By synthesizing a series of block copolymers with various chemical compositions, we demonstrate fabrication of a series of NPs with easily modulated physicochemical properties, which may be themselves be useful for applications in drug delivery.

### Polymer–Nanoparticle Hydrogel Shear Rheological Properties

**Figure 3:**
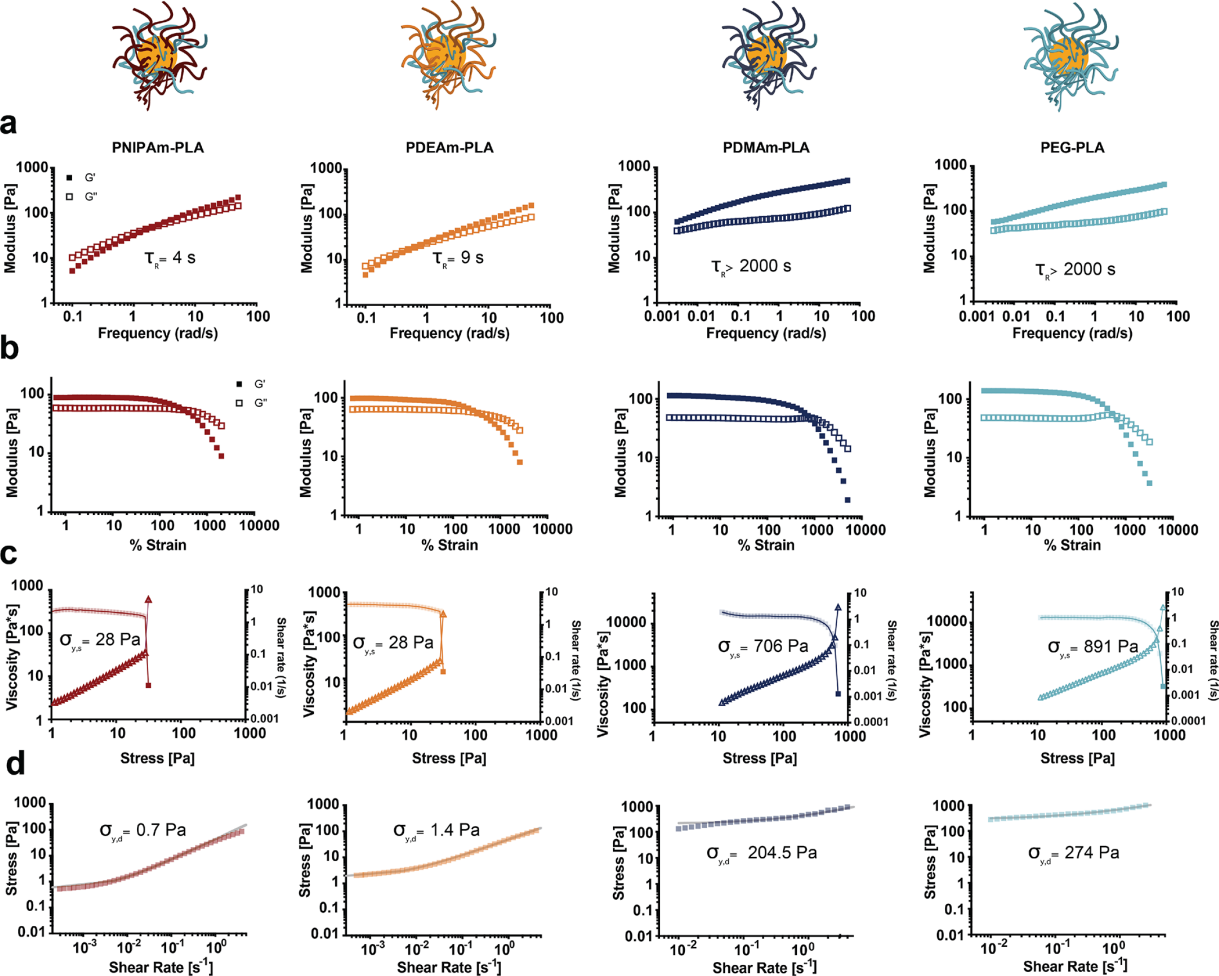
Representative shear rheology of PNP hydrogels consisting of PNIPAm–PLA, PDEAm–PLA, PDMAm–PLA, and PEG–PLA nanoparticles. (a) Frequency sweeps conducted at 1% strain. Relaxation times calculated from the reciprocal crossover of moduli (with units adjusted) denoted on graphs. (b) Amplitude sweeps conducted at 10 rad/s. (c) Static yield stress determined via stress-controlled flow sweep with increasing stress. Viscosity falls at the yield stress (squares, left axis), and the shear rate rises at the yield stress (triangles, right axis). The viscosity before yielding is an apparent preyield viscosity (reduced opacity data points) that becomes a true viscosity measurement upon yielding. Static yield stress values denoted on the corresponding graphs. (d) Dynamic yield stress determined via shear-rate controlled flow sweep, which demonstrates Herschel–Bulkley yielding behavior. Dynamic yield stress values are found through fitting to the Herschel–Bulkley equation (fit in gray) and denoted on the corresponding graphs. Other corresponding Herschel–Bulkley parameters are as follows. PNIPAm–PLA: *n* = 0.80, *K* = 43.2; PDEA–PLA: *n* = 0.64, *K* = 47.1; PDMA–PLA: *n* = 0.65, *K* = 258; PEG–PLA: *n* = 0.55, *K* = 408, where *n* is the shear-thinning index and *K* is the consistency index.

With a series of various NPs in hand, we then formulated PNP hydrogels with a high water content (93% phosphate-buffered saline) by simply mixing with HPMC-C_12_. All of the polycrylamide-based NPs evaluated were formulated with 50% PEG–PLA and 50% polyacrylamide–PLA copolymer. The effects of NP chemistry on the viscoelastic, yielding, and flow properties of the resulting PNP hydrogels were characterized via shear rheology (Figure 3). Viscoelastic moduli can be measured within the linear viscoelastic regime below the yield stress (Figure S4).^2−4,40^ While PNP hydrogels comprising hydrophobic PNIPAm- and PDEAm-based NPs exhibited measurable crossovers between the shear storage and loss moduli on the frequency spectra, and thus short relaxation time scales, the PNP hydrogels comprising hydrophilic PDMAm- and PEG-based NPs did not present a crossover even at very low frequencies. Indeed, the materials comprising PDMAm- and PEG-based NPs exhibited relaxation times longer than those measurable at the lowest torques accessible on a TA Instruments HR-30 rheometer. Notably, while these various PNP hydrogel formulations do not exhibit large differences in stiffness, the changes in NP composition lead to relaxation times that differ by roughly 3 orders of magnitude (Figure S5). Amplitude sweeps varying the strain suggest that all four formulations exhibit yielding at high strains (>300%) (Figure 3b). PNP hydrogels comprising PDMAm- and PEG-based NPs substantially exhibited the Payne effect, denoted by the appearance of a characteristic increase in *G*″ during yielding, which is a trademark of yield stress fluid behavior.^41,42^ PNP hydrogels formulated with NPs comprising 100% PDMAm–PLA, rather than a 1:1 (w:w) mixture of PDMAm–PLA and PEG–PLA, also exhibited robust hydrogel formation and similar rheological characteristics to the PNP hydrogels comprising the other hydrophilic NPs (Figure S6).

In addition to oscillatory testing, flow testing was also performed to assess static and dynamic apparent yield stress behavior in these materials. The static yield stress is defined as the stress at which the material begins to flow upon an increase in stress, while the dynamic yield stress is the minimum stress required for maintaining flow.^43,44^ PNP hydrogels comprising hydrophobic PNIPAm- and PDEAm-based NPs exhibited lower static yield stress behavior than gels comprising hydrophilic PDMAm- and PEG-based NPs. Similarly, the dynamic yield stress values observed for materials comprising hydrophobic NPs were much lower than those of materials comprising hydrophilic NPs, but all PNP hydrogels demonstrated Herschel–Bulkley behavior characteristic of yield stress fluids with clear plateaus at low shear rates.^3,45^ In all materials, the static yield stress was much higher than the dynamic yield stress, suggesting these materials are thixotropic and have a time scale associated with microstructural reformation.^43,44^ Because of these time-dependent effects, both measurements should be considered apparent yield stress values. Critically, this apparent yield stress behavior is only observed in PNP hydrogels, as the HPMC-C_12_ solutions alone do not exhibit prominent yield stress behavior (Figure S7). These findings suggest that the hydrophobicity of the NP corona greatly affects the polymer–nanoparticle interactions with the HPMC-C_12_, providing an avenue for facile modulation of the macrostructural properties and performance of PNP hydrogels.

### Examining Extensibility

To assess whether these formulations might enable unique mechanical properties, we performed filament stretching extensional rheology (fiSER).^15,46−49^ Dilute solutions of cellulose-based polymers have been previously found to exhibit unique extensional behavior.^50,51^ We hypothesized that PNP hydrogels, which comprise cellulose-based polymers, might exhibit high degrees of extensibility compared to otherwise comparable hydrogels based on different biopolymers such as alginate or hyaluronic acid. fiSER is the ideal extensional testing method for these hydrogels due to their high viscosity and viscoelasticity as compared to other recently developed extensional testing methods such as drop-on-substrate analysis (DoS) or capillary breakup extensional rheometry (CaBER), which are limited to lower viscosity and faster relaxing fluids.^50,52−54^ During fiSER testing, the strain-to-break is measured as the percent imposed nominal strain (engineering strain) at which the filament cohesively fails during extensional deformation at a constant Hencky strain rate.^15,55^ The protocol results in an unstable filament with an upper limit of the engineering strain, in contrast to other developed protocols involving devices with feedback control.^55,56^ Strain rates in these experiments were selected to span a wide range to capture any potentially rate-dependent extensional behavior. The strain-to-break is an extrinsic material property, so it is geometry-dependent. We utilized an aspect ratio of 1 to be consistent with previous works (Figure S8).^2,15,46^

**Figure 4:**
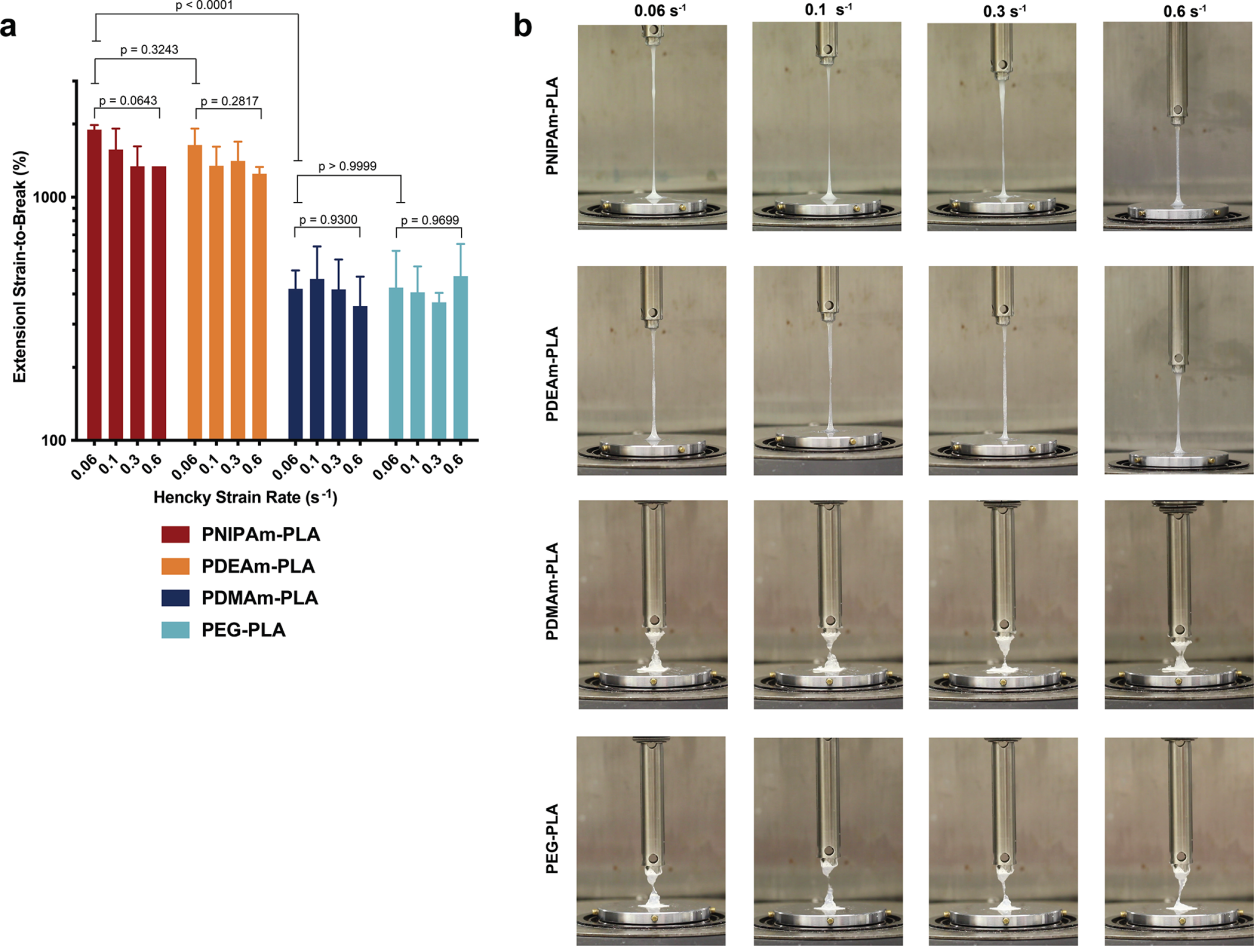
(a) Quantified extensional strain-to-break measurements for various PNP hydrogels (PNIPAm–PLA, PDEAm–PLA, PDMAm–PLA, and PEG–PLA) at varying strain rates. Three separate material replicates were made for each hydrogel formulation. *P* values are calculated with a one-way ANOVA followed by post hoc Tukey multiple comparisons test. (b) Representative images of various PNP hydrogels at varying strain rates directly preceding breaking point. To avoid adhesive failure, a very thin adhesive tape (<0.1 mm thickness) was used on the geometry and Peltier plate in certain formulations.

The fiSER results indicate that PNP hydrogels comprising hydrophobic PNIPAm- and PDEAm-based NPs reach nearly 2000% extensional strain, extending almost 20 times their initial strain (Figure 4 and Table S8). In contrast, PNP hydrogels comprising hydrophilic PDMAm- and PEG-based NPs reached more modest strain-to-break values (∼400%) that are nevertheless still very high compared to other physically cross-linked hydrogel materials.^15^ Filaments from PNP hydrogels comprising hydrophilic NPs broke in more heterogeneous, nonuniform fashions compared to those comprising hydrophobic NPs, which exhibited continuous filament-stretching behavior (Video S1). Additionally, all formulations exhibited minimal strain-dependent changes, with the more hydrophobic PNIPAm- and PDEAm-based NPs exhibiting slight reductions with increased strain rates. It is important to note that these materials are formulated with 93% water content, which is significantly higher than previously published physically cross-linked hydrogel materials exhibiting high extensibility.^8,15,49,57^ Stress data during extension demonstrated that the PNP hydrogels containing hydrophilic NPs reached higher stress values with deformation, approaching their yield stresses, but then dramatically failed (Figure S9). In contrast, PNP hydrogels containing hydrophobic NPs gradually dissipate stress well above their yield stress values during elongation.

In addition to these formulations, comprehensive testing of several PNP hydrogels with varying amounts of PEG–PLA NPs and HPMC-C_12_ polymer solutions were examined and did not exhibit highly extensible behavior (Figures S10 and S11). These results suggest that increasing the concentration of the HPMC-C_12_ polymer increases the PNP hydrogel extensibility, but an increase in PEG–PLA nanoparticles leads to slight reductions in extensibility across strain rates. Previously reported findings show that increasing the HPMC-C_12_ content at a constant PEG–PLA content yields PNP hydrogels with increased liquidlike behavior.^58^ However, polymer solution controls comprising HPMC-C_12_ or HPMC alone exhibit reduced extensibility and greatly increased strain-rate-dependent behavior (i.e., increased strain-to-break with increased rate). Additionally, PNP hydrogels formulated with PNIPAm–PLA NPs and unmodified HPMC polymer showed only moderate extensibility (∼500%), far from the extreme extensible behavior exhibited by materials comprising PNIPAm–PLA NPs and HPMC-C_12_ polymers (Figure S12).

### Tunability of Polymer–Nanoparticle Interactions

**Figure 5:**
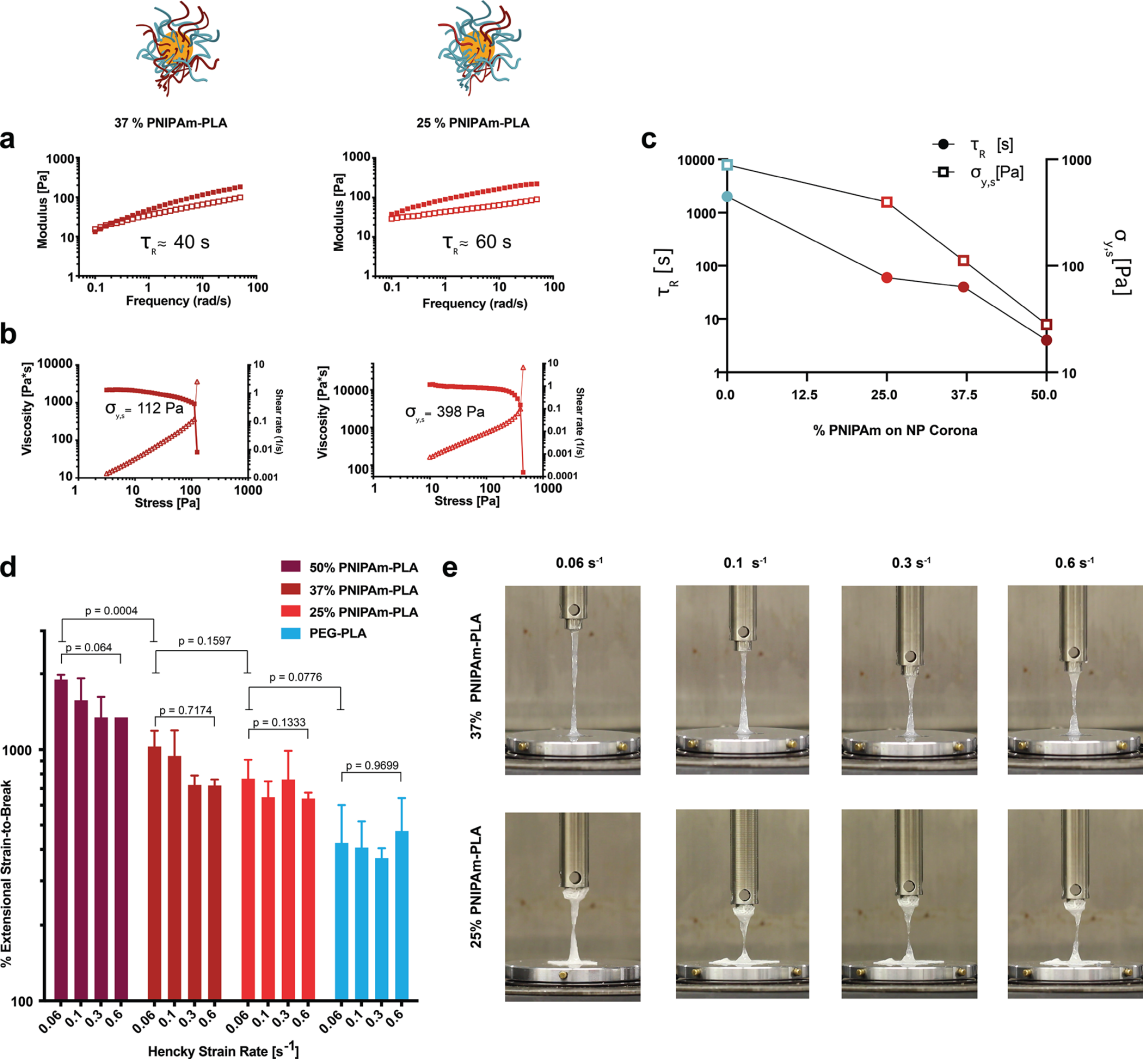
(a) Frequency sweeps conducted at 1% strain of PNP hydrogels comprising NPs with varying amounts of PNIPAm–PLA (37% and 25%; remaining mass within the NPs being PEG–PLA). (b) Stress-controlled flow sweeps of these same PNP hydrogel formulations demonstrating the dramatic drop in viscosity at the static yield stress (squares, left axis) and rise in the shear rate (triangles, right axis). (c) Plot demonstrating the impact of PNIPAm content in the NPs on the relaxation time scale and yield stress of the resulting PNP hydrogels. (d) Extensional strain-to-break measurements at various strain rates for PNP hydrogel formulations comprising NPs with decreasing PNIPAm–PLA content in NPs. Three separate material replicates were obtained for each strain rate. *P* values are calculated with a one-way ANOVA followed by a post hoc Tukey multiple comparisons test. (e) Representative images of the strain-to-break of PNP hydrogels comprising NPs with varying amounts of PNIPAm–PLA.

In addition to tuning the physical properties of PNP hydrogels through alteration of the NP corona composition, we explored the effect of the density of hydrophobic polymer within the NP corona. NPs were prepared with varying PNIPAm–PLA content (50%, 37%, and 25%), where the remaining mass was PEG–PLA. Shear rheology of the resulting PNP hydrogels demonstrated that alteration of the PNIPAm content in the NP corona modulates the relaxation and yield stress behavior (Figure 5a–c). Additionally, fiSER experiments showed that these materials exhibit increased extensibility with increased PNIPAm content in the NP corona (Figure 5d,e and Table S8). These findings suggest the mechanics of this system are highly tunable through simple alteration of the composition of the NPs and the resulting changes to the dynamic PNP cross-linking interactions.

### Probing Recovery Time Scales of PNP Hydrogels

We hypothesized that the increased extensibility of the hydrogels was associated with the bulk relaxation behavior of the PNP hydrogels allowing the continuous dissipation of stress as the strain increased; however, we had not yet examined if recovery rate in this material may be playing a key role. We aimed to identify if the change in relaxation time scales was more crucial to these exceptional mechanical properties.

**Figure 6:**
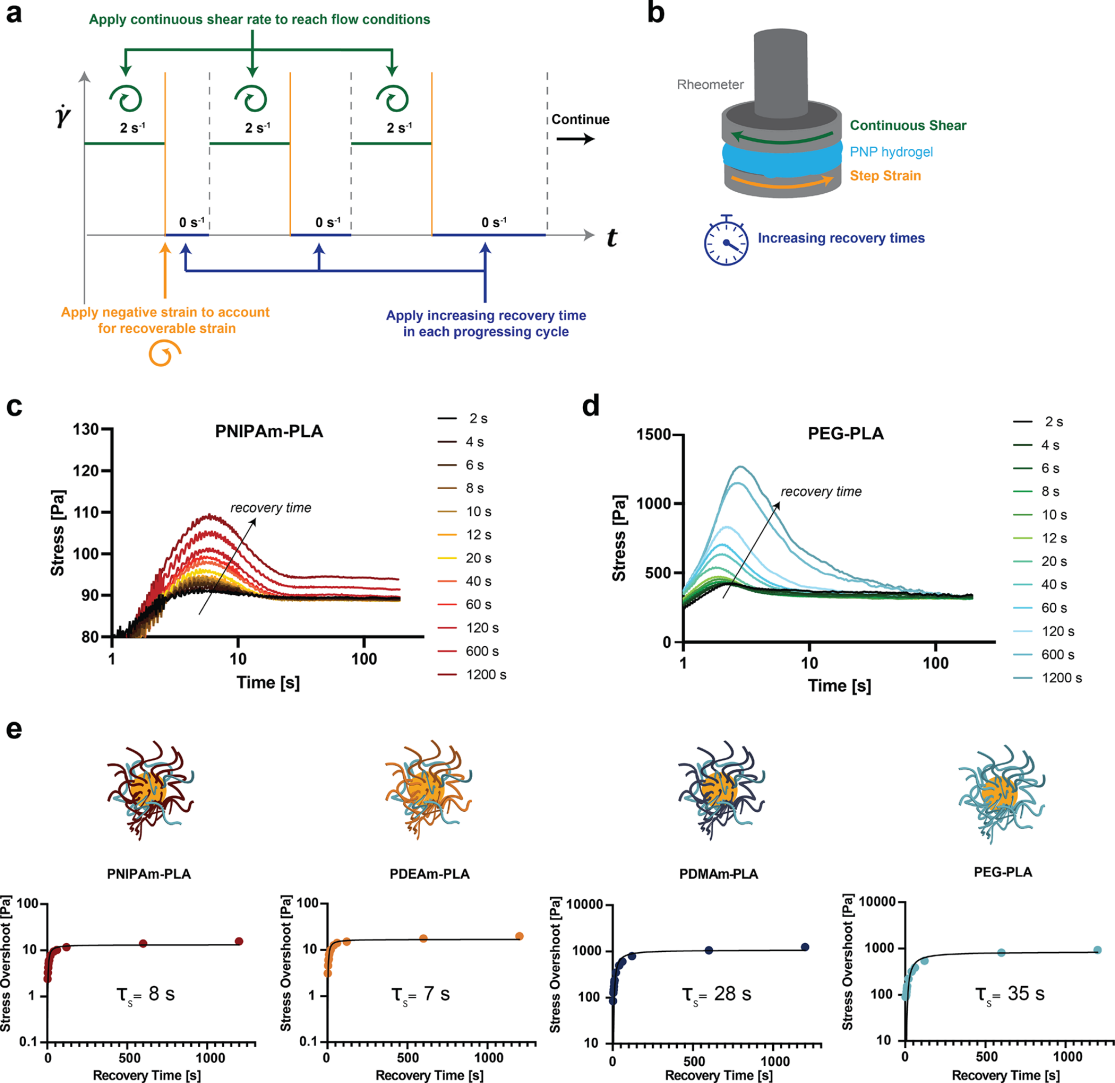
(a) Diagram describing the experimental protocol implemented for the recovery analysis. Recoverable strain measurements are shown in Figure S13. (b) Schematic illustrating the steps of the experimental protocol implemented for the recovery analysis. Stress overshoot measurements with varying recovery times between imposed shear for PNP hydrogels comprising NPs made with (c) PNIPAm or (d) PEG coronas. (e) Stress overshoot data with varying recovery times for the entire series of PNP hydrogels with exponential fit shown in gray. Recovery times based on exponential fit denoted in the corresponding graphs.

To examine the time scale associated with recovery, or thixotropy, we performed stress-overshoot experiments on all formulations at shear rates in the flow regime.^59^ Stress overshoot behavior, while not fully understood, has been found to be characteristic of yield stress fluid behavior.^60,61^ The stress overshoot during shear was measured after various wait times while compensating for a measured recoverable strain after yielding between each step (Figure 6a and Figure S13).^59^ McKinley and co-workers reported that the growth of this overshoot is representative of a characteristic recovery or restructuring time scale (Figure 6c–e).^62^ We find that an analogous time scale (τ_S_) can be found by fitting the stress overshoot growth data according to
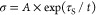
1where σ is the stress overshoot, *t* is the wait time, and *A* is a scaling constant.

PNP hydrogels made with hydrophobic PNIPAm- and PDEAm-based NPs exhibited distinct restructuring behavior from those made with hydrophilic PEG- and PDMAm-based NPs, but both demonstrated a time-dependent restructuring consistent with our static and dynamic yield stress analysis. The magnitudes of the stress overshoot were much smaller for PNP hydrogels comprising hydrophobic PNIPAm- and PDEAm-based NPs (Figure 6a,b), consistent with the reduced yield stress behavior observed for these materials. Moreover, the restructuring time scales obtained by fitting of the stress overshoot growth data were also much shorter for the hydrogels comprising these hydrophobic NPs, commensurate with the observations described above. In contrast, PNP hydrogels comprising hydrophilic PEG- and PDMAm-based NPs exhibited long time scales for complete restructuring as well as large magnitude stress overshoot behavior at long wait times. Consistent with our previous findings, PNP hydrogels comprising NPs made with varying amounts of PNIPAm in the corona exhibited tunable recovery time scales and restructuring behavior (Figure S14).

### Examining the Origins of Extreme Extensibility

Our findings thus far suggest that the incorporation of PNIPAm and PDEAm into the corona of the NP structural motifs within PNP hydrogels leads to both shorter relaxation time scales and shorter recovery time scales. The dynamics of the interactions between the HPMC-C_12_ and the NPs in these materials are faster and can be precisely controlled by tuning the content of hydrophobic polymer in the NP corona. In addition to these two material property time scales, we also performed a stress–relaxation analysis using Kohlrausch’s stretched-exponential relaxation model,^63^ indicating that PNP hydrogels comprising more hydrophobic NPs exhibited shorter stress–relaxation time scales than materials comprising more hydrophilic NPs (Figure S15), commensurate with our other observations.^63,64^

**Figure 7:**
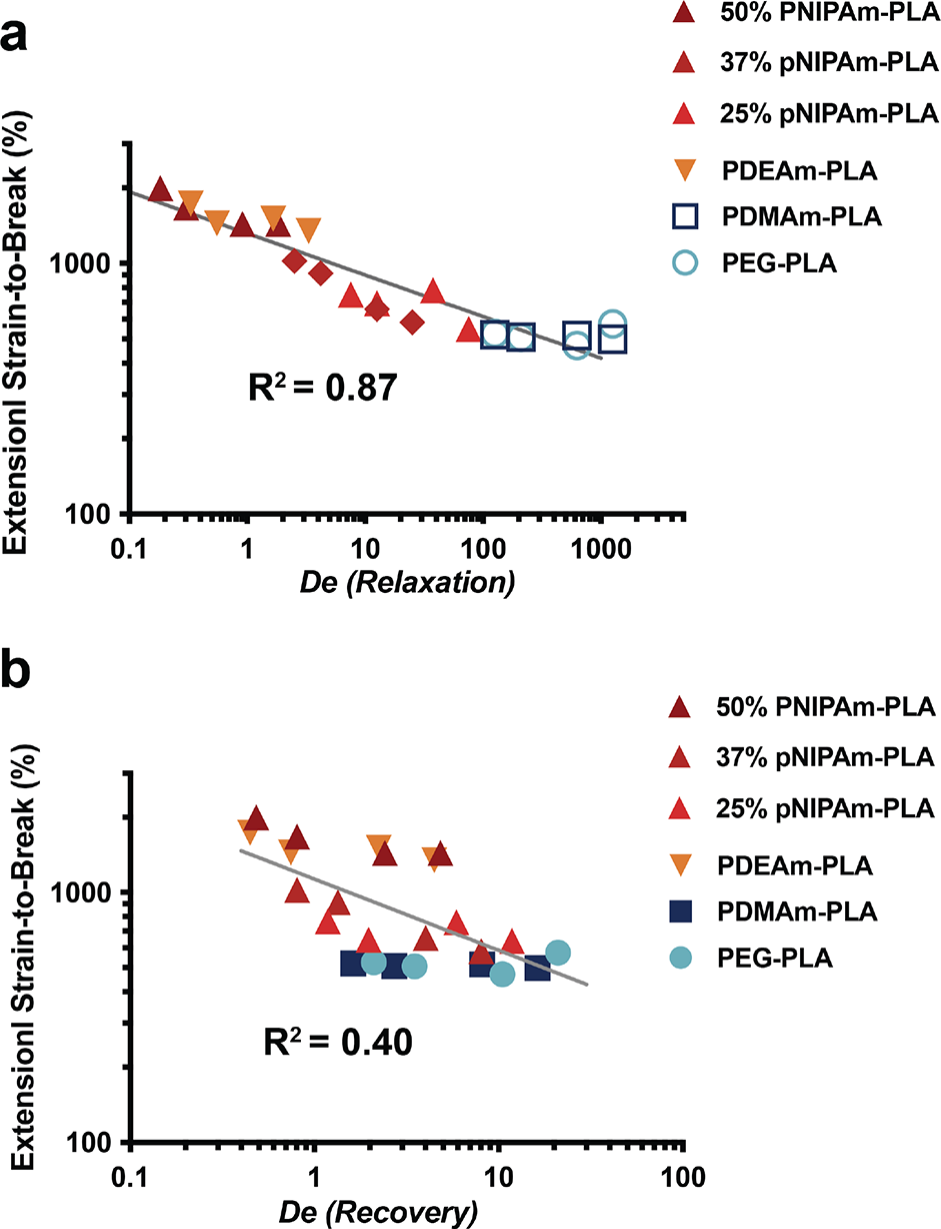
(a) Deborah number, *De*, based on the shear relaxation time and strain rates in extension plotted against the average extensional strain to break for PNP hydrogels. Open symbols represent approximated relaxation time of 2000 s. (b) Deborah number, *De*, based on the shear recovery time and strain rates in extension plotted against the average extensional strain to break for PNP hydrogels. On both plots a log–log linear fit is shown in gray with the *R*^2^ displayed for the corresponding fit that encompasses all replicates from the fiSER experiments.

To probe whether the differences in relaxation or recovery time scales were more indicative of extensibility, we used dimensional analysis to determine an effective Deborah number (*De*) for these processes for each material evaluated. The Deborah number (*De*) is defined as , which represents a ratio of the time scales of interest (τ_R_ or τ_S_) and the time scale of the applied strain rate of our fiSER experiments.^57^ When both the bulk relaxation and recovery time scales are nondimensionalized and plotted against the extensional strain to break, a clear trend emerges with the bulk relaxation time (Figures 7 and S15), suggesting that the faster relaxation dynamics of PNP hydrogels comprising more hydrophobic NPs play a role in increasing the extensibility of these materials at these time scales. While the bulk relaxation time scale trends with extensibility, it is possible that structural changes arising from incorporation of the modified NPs may occur that also affect the extensibility. Notably, HPMC-C_12_ polymer solutions with very fast relaxation times (*De* < 0.1) did not exhibit high extensibility (Figure S11). Relaxation times that are too rapid likely lead to the fluid flowing faster than the extensional time scale and thereby resulting in reduced extensibility. Thus, there may be an optimal Deborah number for a given extensional time scale, as we observe in this study.

The PNP hydrogels comprising hydrophobic PNIPAm- and PDEAm-based NPs allow us to uniquely access improved extensibility in these materials, while still maintaining their yield stress behavior. These hydrophobic polymers likely form a more compact (i.e., less extended and hydrated) corona on the NPs than the hydrophilic polymers do, thereby reducing the interaction between the NPs and the HPMC-C_12_. These trends indicate that modulation of the relaxation behavior (i.e., *De* according to relaxation time scales) can be used to design yield stress fluids to meet application-specific material properties.^15^ These findings are highly relevant to fields such as 3D bioprinting or fiber spinning, where extrusion fidelity is highly related to the extensible characteristics of the material.^2^

## Conclusion

**Figure 8:**
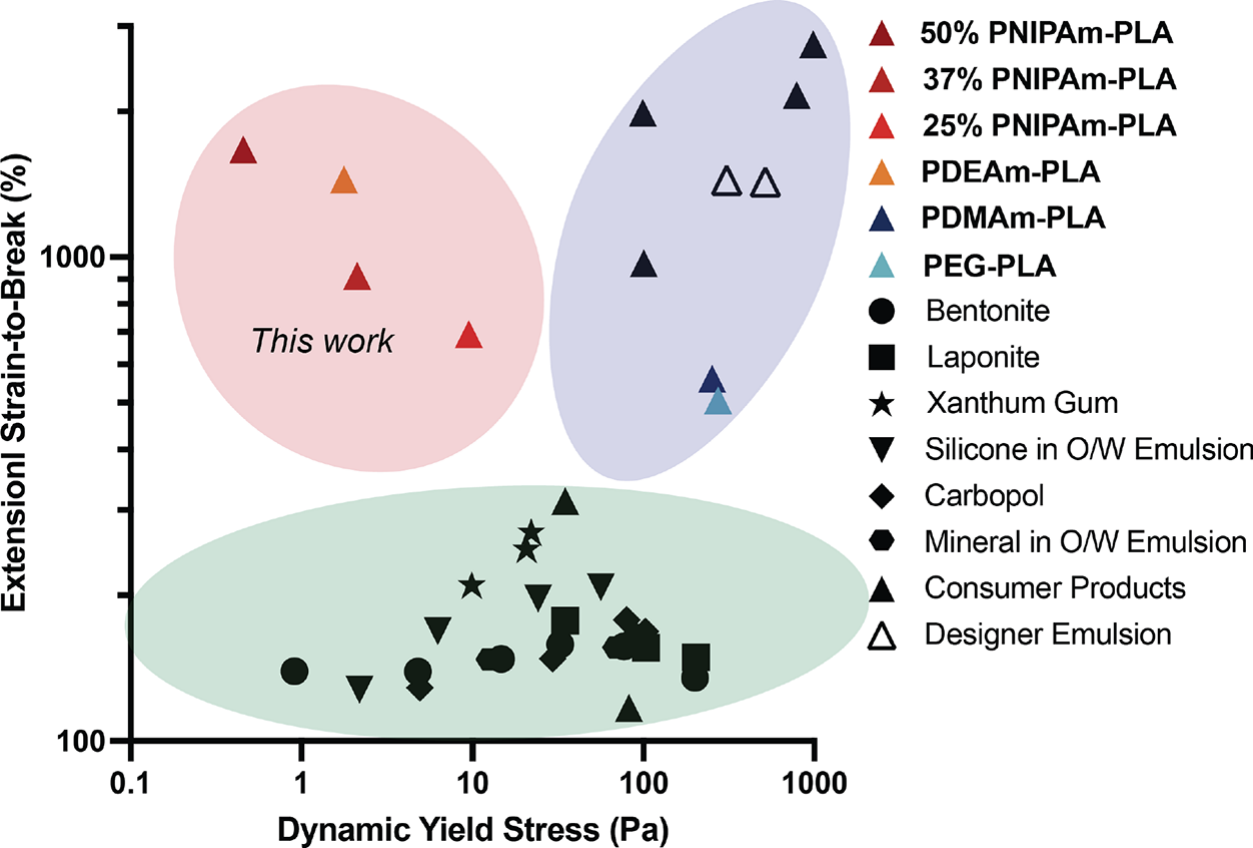
Ashby style plot of the materials space of dynamic yield stress and extensional strain to break. Three clear regions emerge in this space: (i) the green oval represents standard yield stress materials,^15^ (ii) the purple oval represents designer materials^2,15,57,65^ and consumer products^15^ with high yield stress behavior and high extensibility, and (iii) the red oval represents a new design space enabled by this work with materials exhibiting low yet measurable yield stress behavior and high extensibility. Data shown with black symbols are reproduced from Nelson et al.^15^

By leveraging distinctly strong yet dynamic polymer–nanoparticle interactions between biodegradable NPs and hydrophobically modified HPMC polymers, we have developed highly extensible and notably high water content (93%) physically cross-linked yield stress fluids. Indeed, we find that, in comparison to current yield stress fluids of interest, particularly several biomaterials commonly used in various biomedical applications, these PNP hydrogel materials enable access to entirely new combinations of extensibility and yield stress (Figure 8). We have shown that we can generate materials with low yield stress values (<10 Pa) that are extremely extensible (nearly 2000% strain to break) and that it is possible to precisely tune the degree of extensibility by simply altering the composition of the nanoparticles used in PNP hydrogel formulation. Tuning of dynamic cross-linking in these physically cross-linked hydrogel materials through facile modulation of NP chemistry constitutes a powerful tool for controlling their viscoelasticity, yielding behavior, and extensibility. Using dimensional analysis of several relevant time scales of material properties, we elucidate relationships between rheological parameters and extensibility, suggesting that faster relaxation times or lower strain rates lead to increased extensibility in these materials. This work provides critical insight into the central design criteria for extensible materials for use in various applications of interest such as 3D printing, adhesives, injectable biomaterials, and foods.

## Materials and Methods

### General

All solvents and commercially available chemicals (Sigma-Aldrich, Biosynth Carbosynth, VWR) were used as received unless otherwise stated. Reactions requiring anhydrous conditions were conducted with dry solvents under inert atmosphere (nitrogen). Dry dichloromethane (DCM) was obtained from distillation of DCM (Sigma-Aldrich) over phosphorus pentoxide (Sigma-Aldrich, >98%) under N_2_. 2,2′-Azobis(2-methylpropionitrile) (AIBN, Sigma-Aldrich, >98%) and 3,6-dimethyl-1,4-dioxane-2,5-dione (Sigma-Aldrich, 99%) were respectively recrystallized from methanol and ethyl acetate and dried under vacuum. 1,8-Diazabicyclo[5.4.0]undec-7-ene (DBU, Sigma-Aldrich) was distilled before use. NMR spectra were recorded on a Varian 500 MHz spectrometer, and δ values are given in parts per million (ppm).

### THF-SEC-MALLS

The apparent molecular weight and dispersity were determined with the ASTRA software package (Wyatt Technology Corporation) after passing through two size-exclusion chromatography columns (resolve 1000 Å DVB, ID of 7.8 mm, *M*_w_ range of 100–50000 g/mol (Jordi Laboratories); resolve mixed bed low DVB, ID of 7.8 mm, *M*_w_ of range 200–600000 g/mol (Jordi Laboratories)) in a mobile phase of tetrahydrofuran (THF) at 40 °C and a flow rate of 1.0 mL/min. Detection consisted of a Optilab T-rEX (Wyatt Technology Corporation) refractive index detector operating at 658 nm and a TREOS II light scattering detector (Wyatt Technology Corporation) operating at 659 nm. A d*n*/d*c* value of 0.11 for *N*,*N*-dimethylacrylamide in THF was determined in the ASTRA software package by batch injection of four samples of known concentrations into an Optilab T-rEX refractive index detector.

### Synthesis of Dodecyl-Modified HPMC

Hypromellose (HPMC, 1.5 g) was dissolved in 60 mL of anhydrous *N*-methylpyrrolidone (NMP) by stirring overnight at room temperature. Once the polymer had completely dissolved, the solution was heated to at 50 °C for 30 min. A solution of dodecyl isocyanate (0.75 mmol, 183 μL) was dissolved in 5 mL of anhydrous NMP and added to the reaction mixture followed by 105 μL of *N*,*N*-diisopropylethylamine (0.06 mmol). The solution was stirred at room temperature for 20 h. This solution was then precipitated from acetone, and the hydrophobically modified HPMC polymer was recovered by filtration yielding HPMC-C_12_. The polymer was purified through dialysis (3 kDa mesh) in Milli-Q water for 4 days and lyophilized to yield a white amorphous polymer. The ratio of integrations between peaks (δ = 0.8 ppm and δ = 1 ppm) in ^1^H NMR suggests a modification of 8.5 wt %, while the synthetic target was 10 wt % (Figure S1).

### Synthesis of TA-CTA Transfer Agent

According to the literature,^66^ 4-cyano-4-[(dodecylsulfanylthiocarbonyl)sulfanyl]pentanoic acid (10 g, 24.77 mmol, 1 equiv), 2-thiazoline-2-thiol (3.84 g, 32.20 mmol, 1.3 equiv), and 4-(dimethylamino)pyridine (0.42 g, 3.47 mmol, 0.14 equiv) were dissolved in 200 mL of dry DCM. A solution of *N*,*N′*-diisopropylcarbodiimide (4.06 g, 32.20 mmol, 1.3 equiv) in 30 mL of dry DCM was then added at once at 0 °C and reacted for 24 h. The solution was washed twice with 30 mL of water. The product was extracted from an emulsion that formed at the interface via repeated washes with DCM. The organic phase was dried with sodium sulfate and concentrated under reduced pressure. The crude product was purified by flash chromatography (Biotage) on silica gel eluting with pentane/ethyl acetate (3:1), yielding an orange oil which crystallized overnight into an orange solid with poppylike brilliance (8.21 g, 16.3 mmol, 66%). NMR spectroscopic data were in agreement with those previously described.

### Synthesis of Azide-PLA Polymer

2-Azidoethanol (3.0 μL, 4.3 mg, 0.05 mmol) and 1,8-diazabicyclo[5.4.0]undec-7-ene (DBU, 15 μL, 0.1 mmol; 1.4 mol % relative to LA) were dissolved in 1 mL of dry DCM under N_2_. 3,6-Dimethyl-1,4-dioxane-2,5-dione (LA, 1.0 g, 6.9 mmol) was dissolved in 3.5 mL of dry DCM at 40 °C under N_2_. The LA solution was then rapidly added to the first one and was allowed to stir rapidly for 8.5 min under N_2_ at room temperature. The azide-PLA polymer was precipitated from a 50:50 mixture of cold diethyl ether and hexanes and dried under vacuum to yield a white amorphous polymer.

### Synthesis of PNIPAm–PLA Copolymer

#### PNIPAm Synthesis

NIPAm (1.78 g, 53.1 equiv, 15.7 mmol), TA-CTA (0.15 g, 1 equiv, 0.29 mmol), and AIBN (4.8 mg, 0.1 eq, 0.029 mmol) were dissolved in 5.5 mL of DMF in a 20 mL scintillation vial equipped with a PTFE septa. The reaction was sparged with N_2_ for 10 min and heated at 65 °C for 16 h. Monomer conversion (99%+) was determined via ^1^H NMR spectroscopy in CDCl_3_ by the disappearance of vinyl protons (δ = 6.0–6.3 ppm) using DMF as an internal standard. The resulting polymer was precipitated from a 75:25 mixture of ether and hexanes and dried under vacuum. *M*_n_ and dispersity were determined via SEC-MALLS in THF with a d*n*/d*c* of 0.11 determined from the literature.^67^

#### BCN–PNIPAm

PNIPAm (850 mg, 1 equiv, 0.14 mmol) was dissolved in 8 mL of dioxane in a 20 mL scintillation vial. BCN-amine (58 mg, 1.3 equiv, 0.18 mmol) was dissolved in 1 mL of dioxane and transferred to the solution containing PNIPAm. The reaction was closed to air and left at room temperature for 24 h. Successful transamidification was confirmed via ^1^H NMR spectroscopy in CDCl_3_ by the disappearance of TA protons (δ = 4.5 ppm) and appearance of alpha amide protons (δ = 4.1 ppm). The resulting polymer was precipitated from a 75:25 mixture of ether and hexanes and dried under vacuum.

#### PNIPAm–PLA Click Reaction

BCN–PNIPAm (700 mg, 2 equiv, 0.11 mmol) was dissolved in 1 mL of DMF in a 20 mL scintillation vial. Azide-PLA (1100 mg, 1 equiv, 0.055 mmol) was dissolved in 2 mL of DMF and transferred to the solution containing PNIPAm. The reaction was left for 16 h at room temperature (Scheme S1). The resulting copolymer was isolated from unreacted PNIPAm through rapid addition of Milli-Q water and vigorous agitation. The water phase was discarded, and the resulting solid was diluted in dioxane, precipitated into hexanes, and dried under vacuum. *M*_n_ and dispersity were determined via SEC-MALLS in THF with a d*n*/d*c* of 0.056, the arithmetic mean of the d*n*/d*c*s of PLA and PNIPAm.

### Synthesis of PDEAm–PLA Copolymer

#### PDEAm Synthesis

*N*,*N*-Diethylacrylamide (2.40 g, 47.2 equiv, 18.9 mmol), TA–CTA (200 mg, 1 equiv, 0.40 mmol), and AIBN (6.6 mg, 0.1 equiv, 0.04 mmol) were dissolved in 7.4 mL of dioxane in a 20 mL scintillation vial equipped with a PTFE septa. The reaction was sparged with N_2_ for 10 min and heated at 65 °C for 16 h. Monomer conversion (99%+) was determined via ^1^H NMR spectroscopy in CDCl_3_ by the disappearance of vinyl protons (δ = 5.6 ppm) using dioxane as an internal standard. The resulting polymer was precipitated from a 75:25 mixture of ether and hexanes and dried under vacuum. *M*_n_ and dispersity were determined via SEC-MALLS in THF with an approximated d*n*/d*c* of 0.11.^67^

#### BCN–PDEAm

PDEAm (850 mg, 1 equiv, 0.14 mmol) was dissolved in 8 mL of dioxane in a 20 mL scintillation vial. BCN-amine (58 mg, 1.3 equiv, 0.18 mmol) was dissolved in 1 mL of dioxane and transferred to the solution containing PDEAm. The reaction was closed to air and left at room temperature for 24 h. Successful transamidification was confirmed via ^1^H NMR spectroscopy in CDCl_3_ by the disappearance of TA protons (δ = 4.5 ppm) and appearance of alpha amide protons (δ = 4.1 ppm).

#### PDEAm–PLA Click Reaction

PDEAm (700 mg, 2 equiv, 0.11 mmol) was dissolved in 1 mL of DMF in a 20 mL scintillation vial. Azide-PLA (1100 mg, 1 equiv, 0.055 mmol) was dissolved in 2 mL of DMF and transferred to the solution containing PDEAm. The reaction was left for 16 h at room temperature (Scheme S1). The resulting copolymer was isolated from unreacted PDEAm through rapid addition of Milli-Q water and vigorous agitation. The resulting polymer was dissolved into dioxane, precipitated from hexanes, and dried under vacuum. *M*_n_ and dispersity were determined via SEC-MALLS in THF with a d*n*/d*c* of 0.057, the arithmetic mean of the d*n*/d*c*’s of PLA and PDEAm.

### Synthesis of PDMAm–PLA

#### PDMAm Synthesis

*N*,*N*-Dimethylacrylamide (2.4 g, 60.6 equiv, 24.24 mmol), TA–CTA (200 mg, 1 equiv, 0.40 mmol) and AIBN (6.6 mg, 0.1 equiv, 0.04 mmol) were dissolved in 6 mL of dioxane in a 20 mL scintillation vial equipped with a PTFE septa. The reaction was sparged with N_2_ for 10 min and heated at 65 °C for 8 h. Monomer conversion (99%) was determined via ^1^H NMR spectroscopy in CDCl_3_ by comparing the integration of remaining vinyl protons (δ = 5.65 ppm) to the integration of terminal protons on the CTA Z group (δ = 0.8 ppm). The resulting polymer was precipitated from a 75:25 mixture of ether and hexanes and dried under vacuum. *M*_n_ and dispersity were determined via SEC-MALLS in THF with an approximated d*n*/d*c* of 0.11.^67^

#### BCN–PDMAm

PDMAm (617 mg, 1 equiv, 0.10 mmol) was dissolved in 6 mL of dioxane in a 20 mL scintillation vial. BCN-amine (39 mg, 1.2 equiv, 0.12 mmol) was dissolved in 1 mL of dioxane and transferred to the solution containing PDMAm. The reaction was closed to air and left at room temperature for 1 h. Successful transamidification was confirmed via ^1^H NMR spectroscopy in CDCl_3_ by the disappearance of TA protons (δ = 4.5 ppm) and appearance of alpha amide protons (δ = 4.1 ppm). The resulting polymer was precipitated into ether and dried under vacuum.

#### PDMAm–PLA Click Reaction

BCN–PDMAm (480 mg, 2 equiv, 0.08 mmol) was dissolved in 1 mL of DMF in a 8 mL scintillation vial. Azide-PLA (884 mg, 1 equiv, 0.04 mmol) was dissolved in 2 mL of DMF and transferred to the solution containing PDMAm. The reaction was left for 16 h at room temperature (Scheme S1). The resulting copolymer was isolated from unreacted PDMAm through rapid addition of Milli-Q water and vigorous agitation. The resulting copolymer was dissolved into dioxane, precipitated from ether, and dried under vacuum. *M*_n_ and dispersity were determined via SEC-MALLS in THF with a d*n*/d*c* of 0.056, the arithmetic mean of the d*n*/d*c*s of PLA and PDMAm.

### Synthesis of PEG–PLA Copolymer

According to the literature,^58^ poly(ethylene glycol) methyl ether (*M*_n_ 5000, 0.25 g, 4.1 mmol) and DBU (15 L, 0.1 mmol; 1.4 mol % relative to LA) were dissolved in 1 mL of dry DCM under N_2_. LA (1.0 g, 6.9 mmol) was dissolved in 3.5 mL of dry DCM at 40 °C under N_2_. The LA solution was then added rapidly to the first one and was allowed to stir rapidly for 8.5 min at room temperature. The PEG–PLA copolymer was then precipitated from an excess of 50:50 mixture of cold diethyl ether and hexanes and dried under vacuum to yield a white amorphous polymer. *M*_n_ and dispersity were determined via SEC-MALLS in THF with a d*n*/d*c* of 0.047, the arithmetic mean of the d*n*/d*c*s of PLA and PEG.^68,69^

### Nanoprecipitation with Block Copolymers

Polymers were dissolved at 25 or 50 mg/mL in a mixture of acetonitrile and/or DMSO (see Table S2 for nanoprecipitation parameters for each type of nanoparticle). The polymer concentration and solvent ratio was optimized in this process to produce nanoparticles for each polymer composition that has a diameter of ∼40 nm. The polymer solution was added dropwise to water (the antisolvent) spinning at 600 rpm (1 mL of polymer solvent dropped into 10 mL of water). Following nanoprecipitation, nanoparticles were centrifuged in 50 mL Amicon filters with a mesh size of 10 kDa at 4000 rpm at 20 °C for 1 h and 15 min. The concentrated nanoparticles were then removed from the Amicon filter, and the filter was washed with phosphate buffered saline. Nanoparticles reached a concentration between 15 and 20 wt %.

### Characterization of Nanoparticles

For all nanoparticles used in this study dynamic light scattering (DLS) was performed on each batch to confirm all particles were monodisperse (PDI < 0.1) with diameters of ∼40 nm (representative data shown in Tables S2–S5). Multiangle light scattering was also performed to confirm our particles of different compositions were the same size (Table S6).

### Hydrogel Formulation

HPMC-C_12_ was dissolved in phosphate-buffered saline at 6 wt % and loaded into a 1 mL Eppendorf tube. A 15–20 wt % nanoparticle solution was then added to phosphate buffered saline. This dilute nanoparticle solution was added to the HPMC-C_12_. The contents were thoroughly mixed by using a long spatula until homogeneous. The tube was then spun on a table top centrifuge for 10 min to remove bubbles and placed at 4 °C overnight prior to testing. All hydrogels formulated were composed of 2 wt % HPMC-C_12_ and 5 wt % nanoparticles with the remaining mass as phosphate-buffered saline unless otherwise specified.

### General Shear Rheology

Rheological testing was performed by using a 20 mm diameter serrated parallel plate at a 600 μm gap on a stress-controlled TA Instruments DHR-2 rheometer with a solvent trap sealed with water to prevent dehydration unless otherwise specified. All experiments were performed at 25 °C. Frequency sweeps were performed at a strain of 1%. Amplitude sweeps were performed at frequency of 10 rad/s. Stress-controlled flow sweeps were performed from low to high stress logarithmically with steady-state sensing. Steady shear flow sweeps were performed from high to low shear rates logarithmically with steady-state sensing. Duplicates for nearly all samples were performed for each test, and representative data are presented. Frequency sweeps for the PNP hydrogels based on PEG–PLA and (50%) PDMAm–PLA were performed on a TA Instruments HR-30 rheometer that can access lower torque ranges.

### Filament Stretching Extensional Rheology

Strain-to-break measurements were performed on a TA Instruments ARES-G2 rheometer in axial mode with an 8 mm serrated plate geometry. Hencky (exponential) strain rates were applied as described by Nelson et al.^15^ A serrated parallel plate with a radius of *R* = 4 mm and advanced Peltier system bottom plate were used. Samples containing 400 μL were loaded at a gap of *H* = 4 mm, resulting in an aspect ratio of *H*/*R* = 1. The serrated plate helped to ensure the material would stick to the plate. For hydrogels that would not appropriately stick to the serrated plate (adhesive failure), a thin medical adhesive tape (<0.1 mm thick) that promoted absorption into the tape and adhesion to the surfaces was applied to the geometry and the Peltier plate below. All experiments were performed at 25 °C and replicated three times from independent batches of hydrogel. To minimize dehydration, samples were quickly loaded and immediately tested within seconds.

### Stress Overshoot Measurements

To perform stress overshoot measurements, the recoverable strain was first found by applying a constant stress (greater than static yield stress) until the material is fully flowing (shear rate of >1 s^–1^). Subsequently, the stress was reduced to zero, and the strain was recorded during recovery. The recoverable strain was measured after 130 s when the material reached a plateau (Figure S13). In the stress overshoot experiments, a 2 s^–1^ flow rate was applied; next, at the end of the flow step a negative step strain equivalent to the recoverable strain was applied, and then the material was left to rest for a specified wait time (as described in Figure 6).

### Stress–Relaxation Measurements

Stress–relaxation experiments were performed through applying 3% step strain and recording the resulting stress and modulus over time on an Ares-G2 rheometer with a 25 mm serrated plate. To assess stress–relaxation time scales, Kohlrausch’s stretched-exponential relaxation model was used to fit the data, as it has been used for many viscoelastic polymer materials.^63^ A time scale τ_SR_ was determined by using
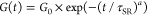
2where *G*_0_ is the plateau modulus, τ_SR_ is the characteristic stress–relaxation time, and *a* is a physical parameter dictated by physical constraints of the system.

## Supporting Information Available

Tables S1–S8 and Figures S1–S15 (PDF)
Video S1 (MOV)

## Supplementary Material

ma2c00649_si_001.pdf

ma2c00649_si_002.mov
