# Supplementary material for: Extreme Extensibility in Physically Cross-Linked Nanocomposite Hydrogels Leveraging Dynamic Polymer–Nanoparticle Interactions
Source: Macromolecules. 2022 Aug 16;55(17):7498–511. doi: 10.1021/acs.macromol.2c00649 (PMC9476865; doi:10.1021/acs.macromol.2c00649)
Supplement: Supplementary file 1 — ma2c00649_si_001.pdf [file ma2c00649_si_001.pdf]

Supporting Information

Extreme Extensibility in Physically Crosslinked Nanocomposite Hydrogels Leveraging Dynamic Polymer-Nanoparticle Interactions

Abigail K. Grosskopf[1], Joseph L. Mann[2], Julie Baillet[2,3], Hector Lopez Hernandez[2], Anton A. A. Autzen[2,4], Anthony C. Yu[2], Eric A. Appel[2,5,6,7,8,*]

1. Department of Chemical Engineering, Stanford University, Stanford CA 94305, USA

2. Department of Materials Science & Engineering, Stanford University, Stanford CA 94305, USA

3.CNRS, Bordeaux INP, LCPO, University of Bordeaux, Pessac, 33600 France

4. Department of Health Technology, Technical University of Denmark, Lyngby, Denmark

5. Department of Bioengineering, Stanford University, Stanford, CA 94305, USA

6. Department of Pediatrics- Endocrinology, Stanford University, Stanford, CA 94305, USA

7. ChEM-H Institute, Stanford University, Stanford, CA 94305, USA

8. Woods Institute for the Environment, Stanford University, Stanford, CA 94305, USA

** denotes corresponding author*

Contents

1 Supplementary Tables **2**

2 Supplementary Figures **10**

3 Supplemental Videos **26**

1 Supplementary Tables

Supplementary Table 1: Mn, Dispersity (Đ), and d*n*/d*c* values of PNIPAm-PLA, PDEAm-PLA, PDMAm-PLA, and PEG-PLA co-polymers.

	Mn	Đ	d*n*/d*c*
PNIPAm-PLA	27400	1.12	0.056
PDEAm-PLA	25900	1.08	0.057
PDMAm-PLA	26500	1.08	0.057
PEG-PLA	22100	1.12	0.047

Supplementary Table 2: Nanoprecipitation parameters to produce monodisperse particles with a diameter of 40 nm for all types of nanoparticles used in this study.

Nanoparticle	Polymer Mass %	Solvent Ratio	Concentration (mg/mL)
PEG-PLA	100% PEG-PLA	65% Acetonitrile, 35% DMSO	50
50% PNIPAm-PLA	50% PNIPAm-PLA, 50% PEG-PLA	100% Acetonitrile	25
25% PNIPAm-PLA	25% PNIPAm-PLA, 75% PEG-PLA	90% Acetonitrile, 10% DMSO	50
37% PNIPAm-PLA	37% PNIPAm-PLA, 63% PEG-PLA	90% Acetonitrile, 10% DMSO	25
PDEAm-PLA	50% PDEAm-PLA, 50% PEG-PLA	100% Acetonitrile	25
PDMAm-PLA	50% PDMAm-PLA, 50% PEG-PLA	50% Acetonitrile, 50% DMSO	50

Supplementary Table 3: Representative dynamic light scattering data from PNIPAm-PLA NPs (a set of 10 acquisitions from the same batch of particles).

Diameter (nm)	Normalized Intensity (Cnt/s)	Radius (nm)	%PD	Mw-R (kDa)	SOS	PD Index	Polydispersity (nm)
42.2	146862249	21.08	0	4216	2.853	4.38E-11	0
46	144346584	23.01	3.8	5176	1.872	3.85E-02	0.9
44.6	140766365	22.32	4	4816	1.294	3.99E-02	0.9
47	144102952	23.48	4.2	5422	2.124	4.15E-02	1
45.2	146063198	22.59	9.3	4956	2.124	9.26E-02	2.1
45.7	142599962	22.85	0	5090	3.319	5.79E-09	0
45.7	151831781	22.83	11.4	5077	2.057	1.15E-01	2.6
43.9	148946345	21.97	10.5	4643	1.553	1.05E-01	2.3
43.3	149932063	21.67	9.2	4497	1.558	9.17E-02	2
43.1	142211880	21.56	18	4440	2.045	1.80E-01	3.9

Supplementary Table 4: Representative dynamic light scattering data from PEG-PLA NPs (a set of 10 acquisitions from the same batch of particles).

Diameter (nm)	Normalized Intensity (Cnt/s)	Radius (nm)	%PD	Mw-R (kDa)	SOS	PD Index	Polydispersity (nm)
41.5	100443469	20.76	0	4068	1.939	2.61E-09	0
41.2	101003942	20.6	8.3	3994	2.007	8.29E-02	1.7
41.1	104067621	20.56	2.8	3974	1.462	2.81E-02	0.6
40.6	101812294	20.29	0	3855	1.665	9.10E-09	0
40	101458451	20	0.8	3728	1.496	8.01E-03	0.2
40.3	100627581	20.16	9.5	3798	1.6	9.52E-02	1.9
40	101256174	20.01	4.2	3731	2.06	4.24E-02	0.8
41.5	98592887	20.75	15.7	4063	1.746	1.57E-01	3.3
39.9	100858053	19.95	17.6	3704	1.453	1.77E-01	3.5
40.4	102882907	20.19	7.1	3812	1.36	7.06E-02	1.4

Supplementary Table 5: Representative dynamic light scattering data from PDEAm-PLA NPs (a set of 10 acquisitions from the same batch of particles).

Diameter (nm)	Normalized Intensity (Cnt/s)	Radius (nm)	%PD	Mw-R (kDa)	SOS	PD Index	Polydispersity (nm)
38.9	133701826	19.43	17.3	3481	0.82	1.73E-01	3.4
38.7	133608210	19.33	8.2	3443	1.657	8.22E-02	1.6
41.8	133177110	20.88	0	4120	2.652	4.75E-10	0
38.9	134620078	19.47	22.2	3501	1.181	2.22E-01	4.3
38.7	131483073	19.33	14.1	3441	1.072	1.41E-01	2.7
39.7	135347595	19.84	14.5	3656	1.716	1.45E-01	2.9
38.3	132679837	19.15	2.2	3365	1.151	2.18E-02	0.4
39.3	129518115	19.64	13.7	3572	1.38	1.37E-01	2.7
39	132003702	19.5	3.7	3513	0.697	3.75E-02	0.7
40.1	131523556	20.07	0	3757	1.388	3.03E-09	0

Supplementary Table 6: Representative dynamic light scattering data from PDMAm-PLA NPs (a set of 10 acquisitions from the same batch of particles).

Diameter (nm)	Normalized Intensity (Cnt/s)	Radius (nm)	%PD	Mw-R (kDa)	SOS	PD Index	Polydispersity (nm)
43.8	91705483	21.88	0	4597	1.563	1.89E-08	0
40.7	89685358	20.35	17.5	3879	2.239	1.75E-01	3.6
45.2	86934992	22.6	3	4963	1.627	2.98E-02	0.7
42.7	87940286	21.37	5.1	4353	1.956	5.12E-02	1.1
42.9	88643280	21.46	12.7	4394	2.09	1.27E-01	2.7
43.9	85944381	21.97	2.3	4643	2.329	2.31E-02	0.5
41.9	87383976	20.97	8.2	4165	2.409	8.16E-02	1.7
42.8	88817666	21.39	15.9	4360	1.905	1.59E-01	3.4
41.8	87644041	20.89	19.1	4125	1.805	1.91E-01	4
42.2	88415004	21.11	0	4231	1.914	1.41E-08	0

Supplementary Table 7: Multi-angle light scattering data from two types of particles to confirm DLS data.

	**Mn (kDa)**	**Mw (kDa)**	**Polydispersity (Mw/Mn)**	**Rn (nm)**	**Rw (nm)**
**PNIPAm-PLA NPs**	8646.1	8989.7	1.04	15.4	15.7
**PEG-PLA NPs**	7297.1	8529.1	1.169	16.7	17.4

Supplementary Table 8: Additional P values for filament stretching experiments from Figures 4 and 5 for various strain rates. P values are calculated with a one-way ANOVA followed by a posthoc Tukey multiple comparisons test.

Group Name	0.06 s^{-1}	0.1 s^{-1}	0.3 s^{-1}	0.6 s^{-1}
PEG-PLA vs. PNIPAm-PLA	<0.0001	0.0011	0.0009	<0.0001
PEG-PLA vs. 37% PNIPAm-PLA	0.004	0.0752	0.1645	0.0366
PEG-PLA vs. 25% PNIPAm-PLA	0.0776	0.5873	0.1171	0.1757
PNIPAm-PLA vs. 37% PNIPAm-PLA	0.0004	0.0391	0.0148	0.0001
PNIPAm-PLA vs. 25% PNIPAm-PLA	<0.0001	0.0047	0.0205	<0.0001
37% PNIPAm-PLA vs. 25% PNIPAm-PLA	0.1957	0.424	0.9944	0.689
PEG-PLA vs. PDEAm-PLA	<0.0001	0.0061	0.0014	0.0001
PEG-PLA vs. PDMAm-PLA	>0.9999	0.9922	0.9917	0.585
PNIPAm-PLA vs. PDEAm-PLA	0.3242	0.6789	0.9758	0.7472
PNIPAm-PLA vs. PDMAm-PLA	<0.0001	0.0022	0.003	<0.0001
PDEAm-PLA vs. PDMAm-PLA	0.0001	0.0087	0.0019	<0.0001

2 Supplementary Figures

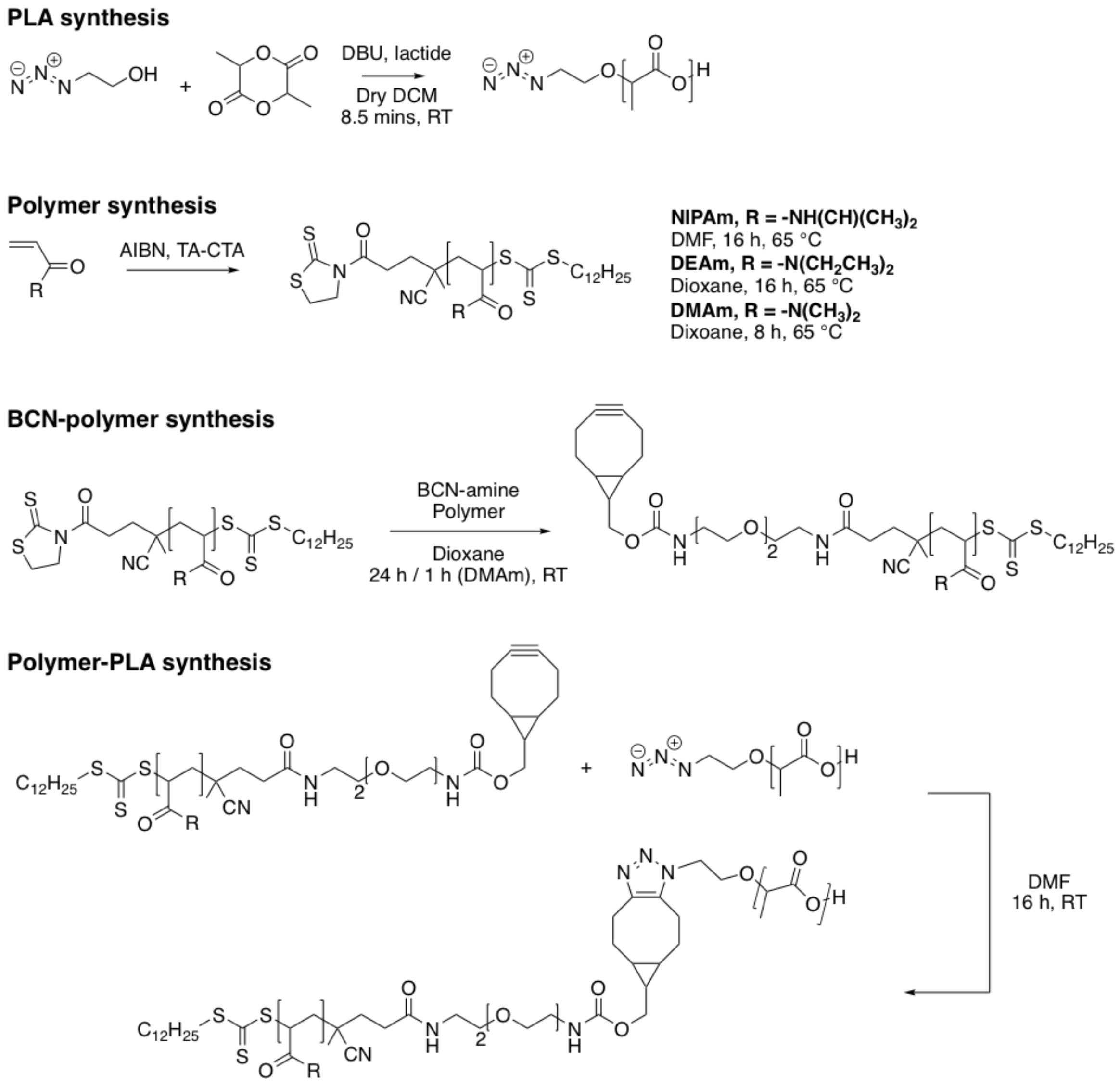

Supplementary Scheme 1: Synthetic route to synthesize PNIPAm-PLA, PDEAm-PLA and PDMAm-PLA.

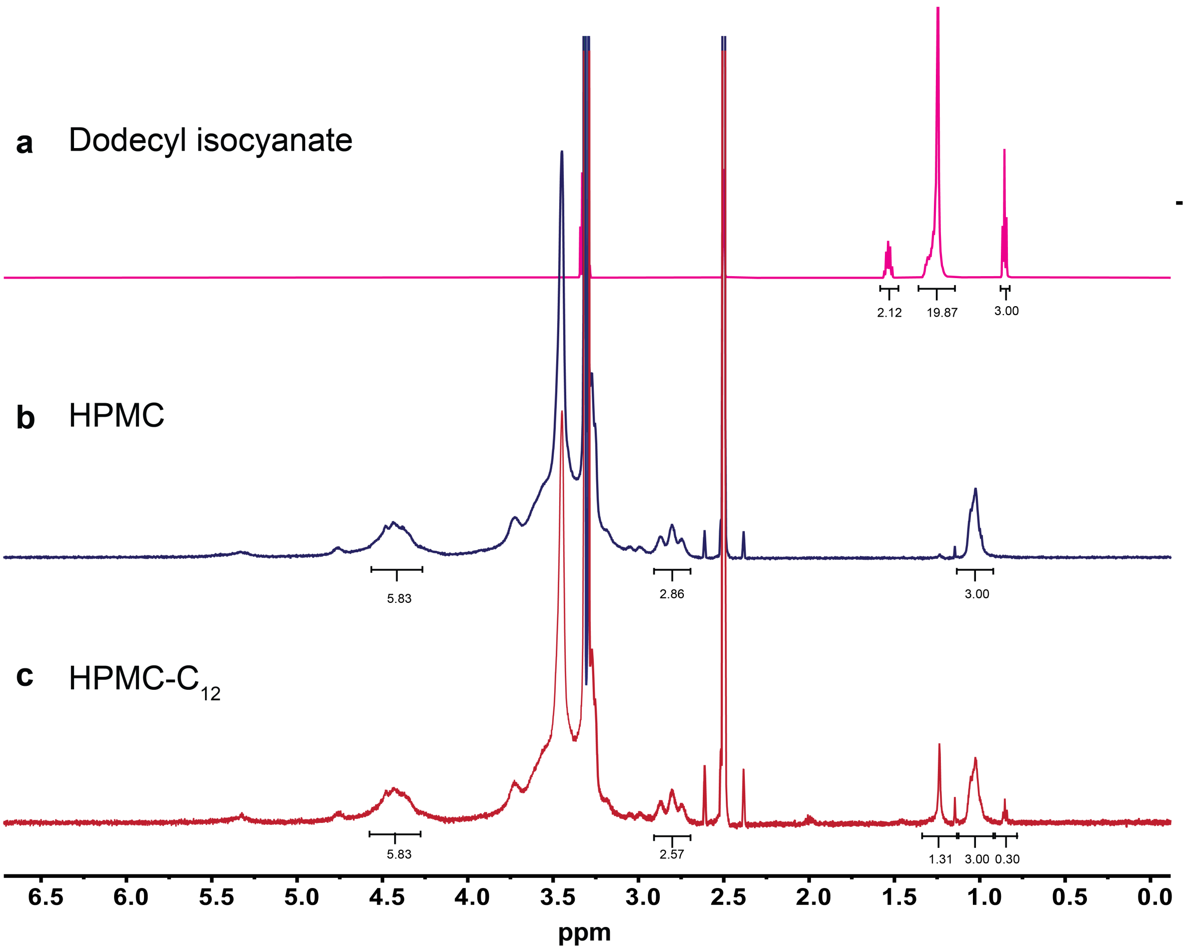

Supplementary Figure 1: ^{1}H-NMR spectra of **a,** Dodecyl isocyanate, **b,** Hypromellose (HPMC), **c,** Dodecyl-modified hydroxypropylmethylcellulose (HPMC-C_{12}) in DMSO-d_6.

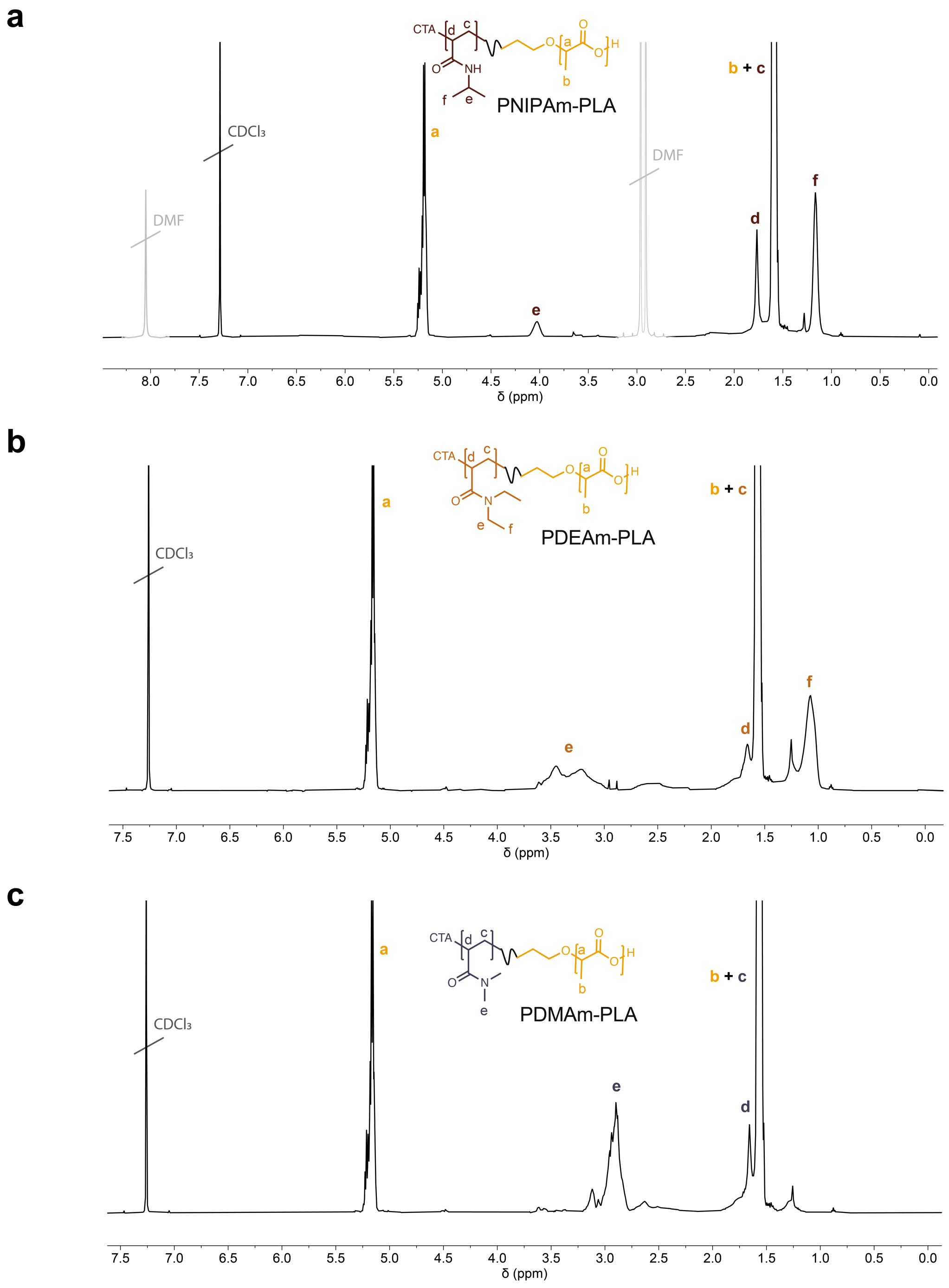

Supplementary Figure 2: ^{1}H-NMR spectra of **a,** PNIPAm-PLA, **b,** PDEAm-PLA, **c,** PDMAm-PLA copolymer in $CDCl_3$.

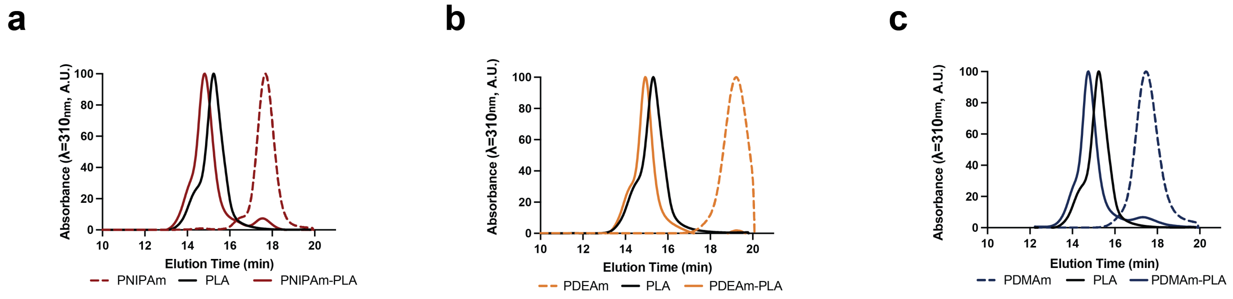

Supplementary Figure 3: SEC traces of the synthesized block copolymers and homopolymer precursors before and after fractional precipitation in water followed by hexanes. **a,** RI traces normalized such that the mp response is identical for the PNIPAm, PLA, and PNIPAm-PLA block copolymer. **b,** RI traces normalized such that the mp response is identical for the PDEAm, PLA, and PDEAm-PLA block copolymer. **c,** RI traces normalized such that the mp response is identical for the PDMAm, PLA, and PDMAm-PLA block copolymer.

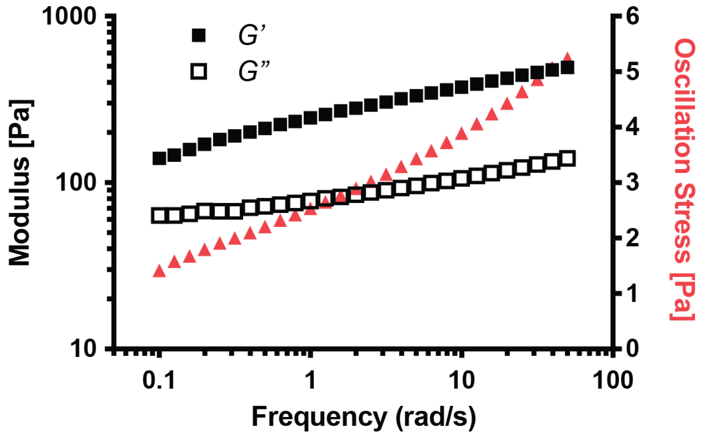

Supplementary Figure 4: Representative frequency sweep within the linear viscoelastic regime of a PEG-PLA based PNP hydrogel demonstrating that the stress does not exceed the yield stress during the experiment and thus the material can be characterized as a viscoelastic solid within this regime.

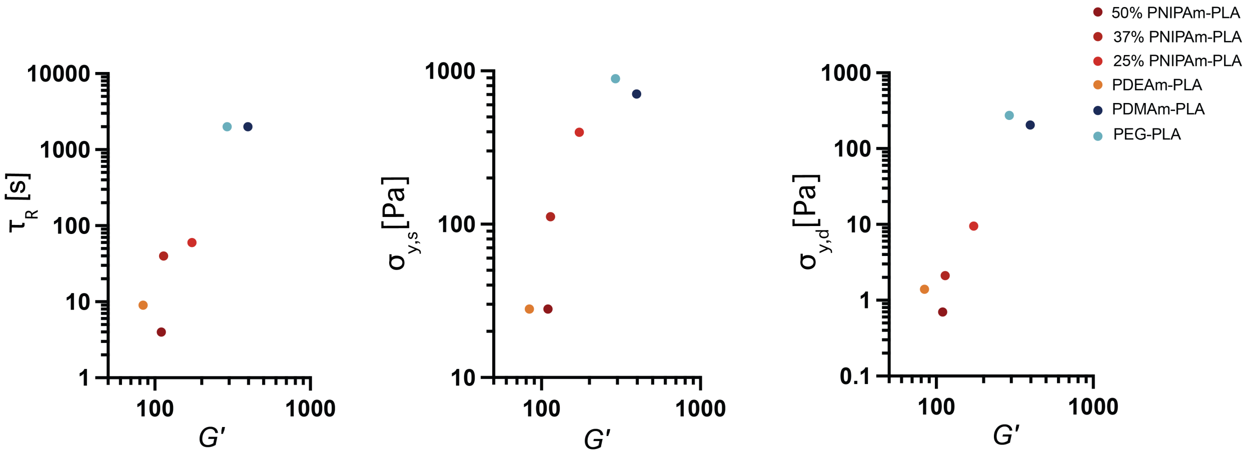

Supplementary Figure 5: Ashby plots mapping PNP hydrogel material properties. The elastic storage modulus *G'* (at 10 rad/s and 1% strain) plotted against the relaxation time τ_R, static yield stress $\sigma_{y,s}$, and dynamic yield stress $\sigma_{y,d}$.

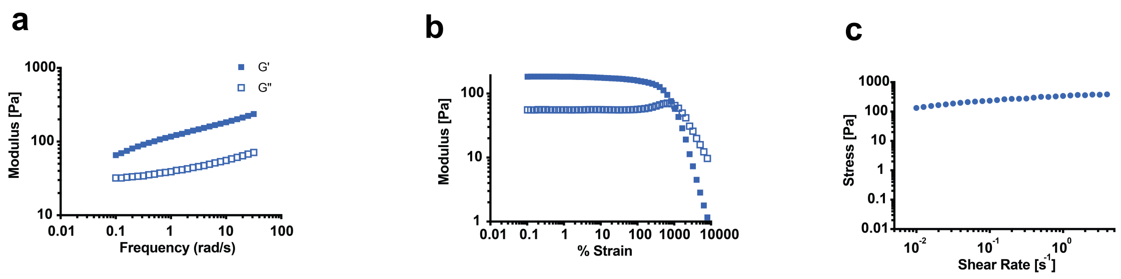

Supplementary Figure 6: Shear rheology of a PNP hydrogel with nanoparticles formulated with 100% PDMAm-PLA (no PEG-PLA). **a,** Frequency sweep at 1% strain. **b,** Amplitude sweep at 10 rad/s. **c,** Dynamic yield stress determined via shear-rate controlled flow sweep.

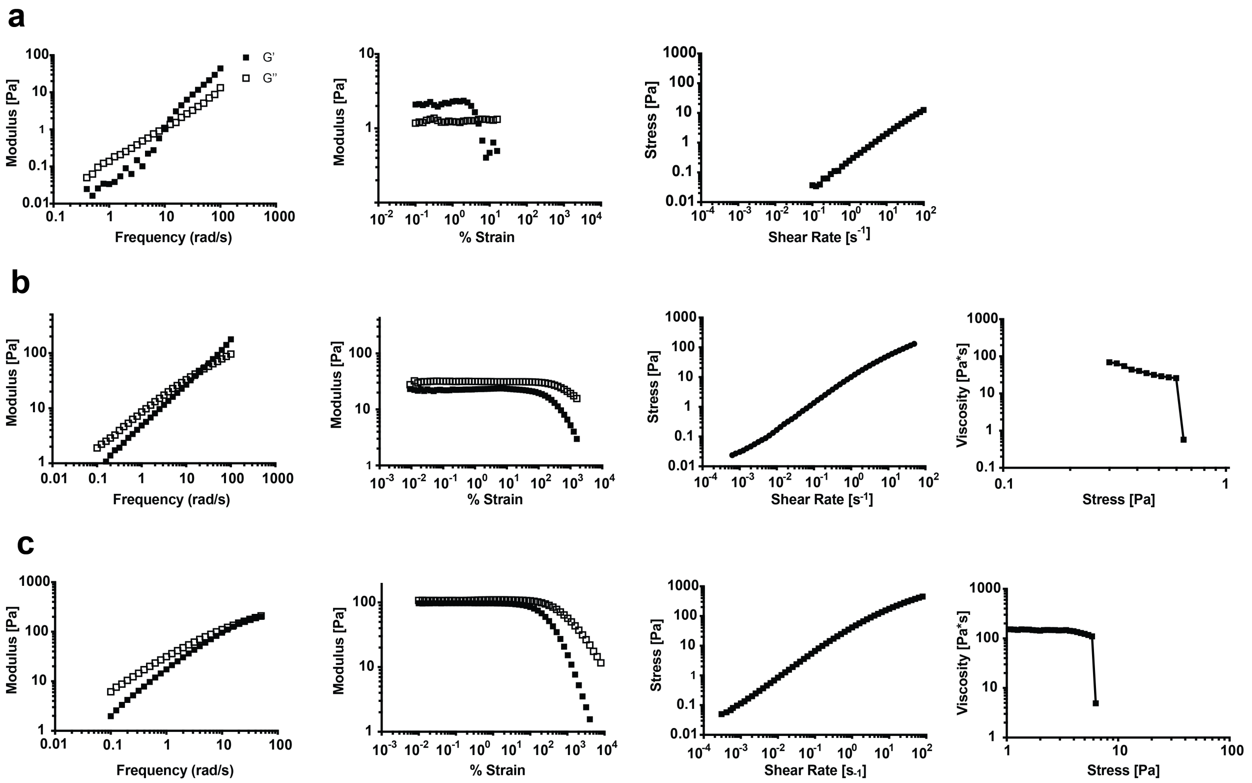

Supplementary Figure 7: Shear rheology of **a,** 1 wt%, **b,** 2 wt%, **c,** 4 wt% HPMC-C_{12} solutions. Frequency sweep at 1% strain, amplitude sweep at 10 rad/s, flow sweep from high to low shear rates with steady state sensing, and stress-controlled flow sweep. The 1 wt% solution is a low viscosity solution did not demonstrate a measurable yield stress in the stress-controlled flow sweep. None of the solutions exhibit any measurable dynamic yield stress behavior.

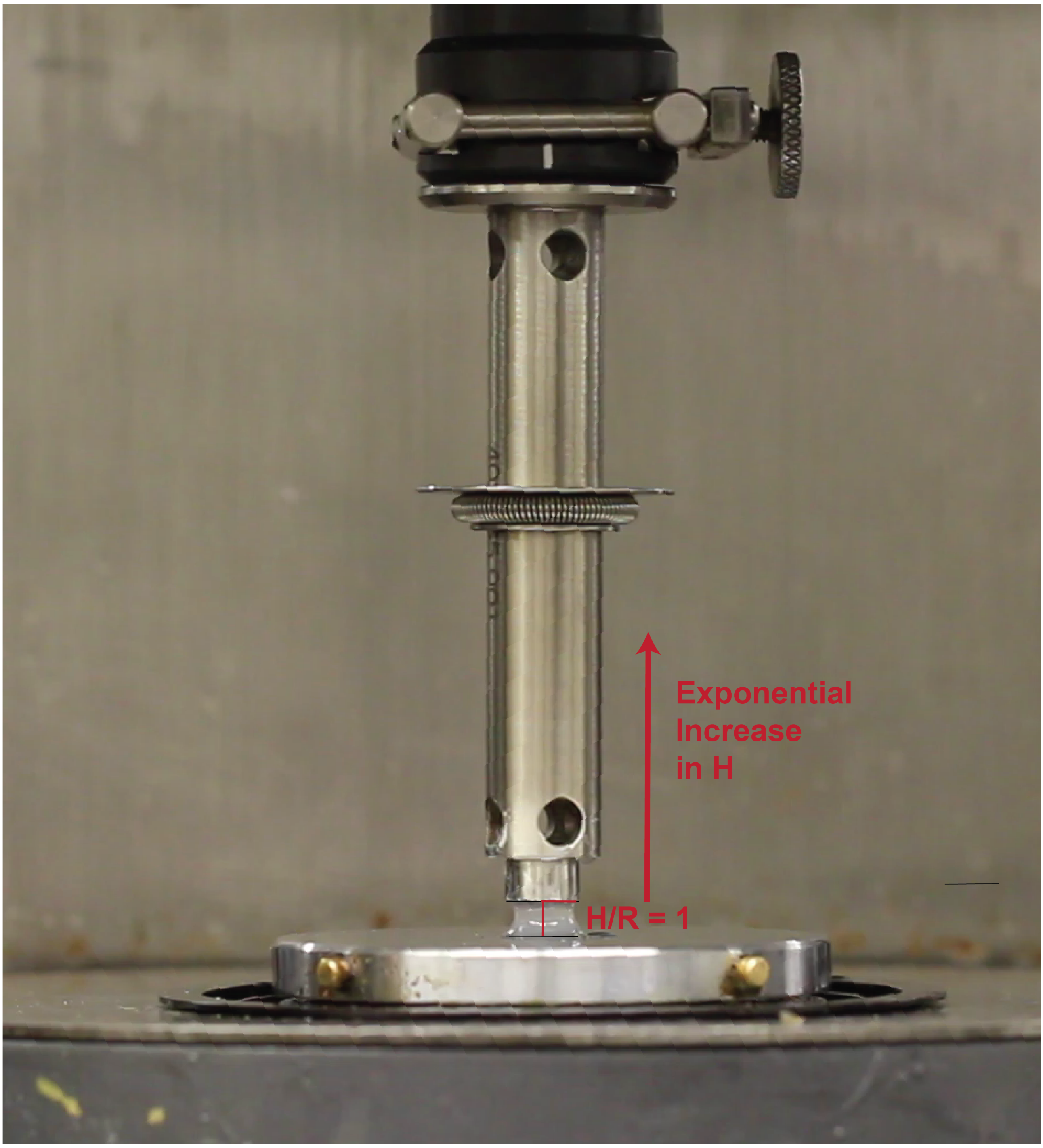

Supplementary Figure 8: Initial set-up for filament stretching rheology experiments. The initial aspect ratio is equal to 1 with a height (H) and radius (R) both equal to 4 mm. An 8 mm diameter serrated parallel plate geometry is used.

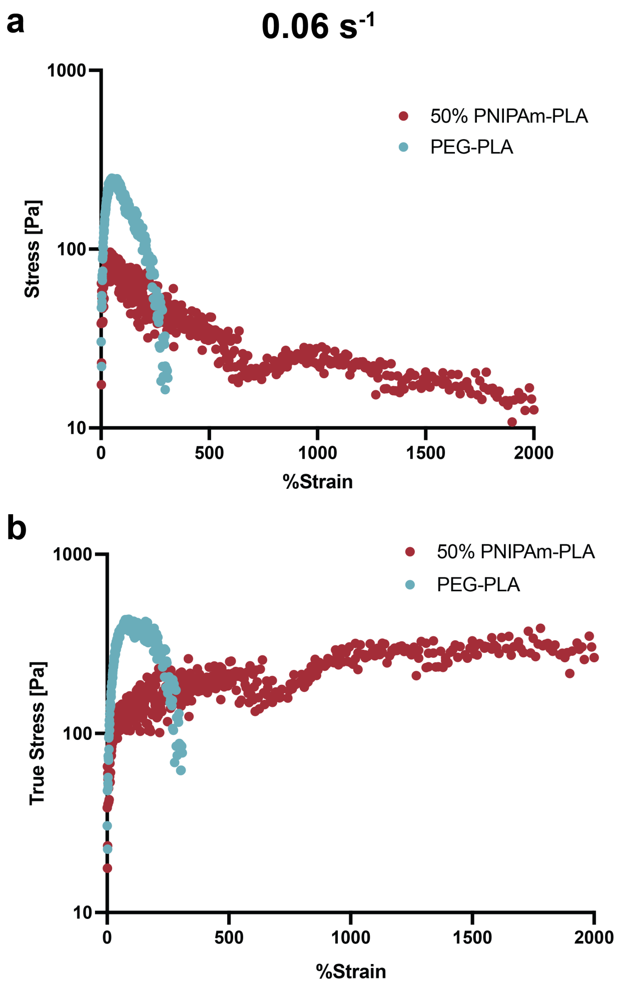

Supplementary Figure 9: Stress-strain data during filament stretching extensional rheology of PNP hydrogels with PNIPAm-PLA and PEG-PLA nanoparticles. **a,** Engineering Stress and **b,** True Stress for strain rates of 0.06 s^{-1} and 0.1 s^{-1}. Due to artifacts in the stress reading due to inertia of the rheometer instrument during movement, the stress-strain data is quite noisy at low strain rates (shown here) and is not observable at higher strain rates, as reported in previously published reports as well.[1]

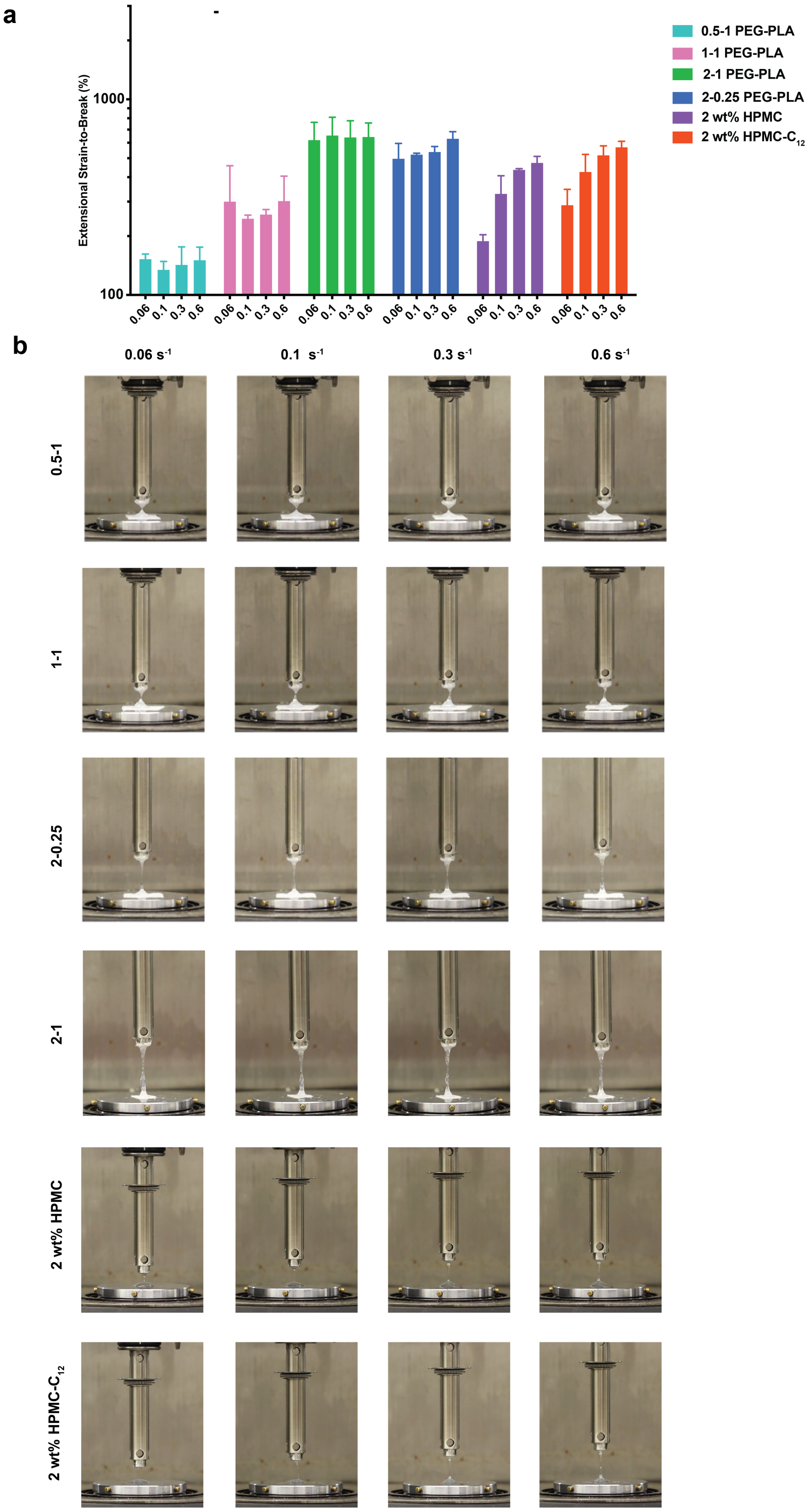

Supplementary Figure 10: Filament stretching extensional rheology of various alternative formulations of PEG-PLA-based PNP hydrogels. The first number represents the wt% of HPMC-C_{12}, while the second number represents the wt% of PEG-PLA nanoparticles (remaining mass is phosphate-buffered saline). **a,** Extensional strain-to-break measurements for all formulations. **b,** Representative images at of all formulations at the strain rates tested.

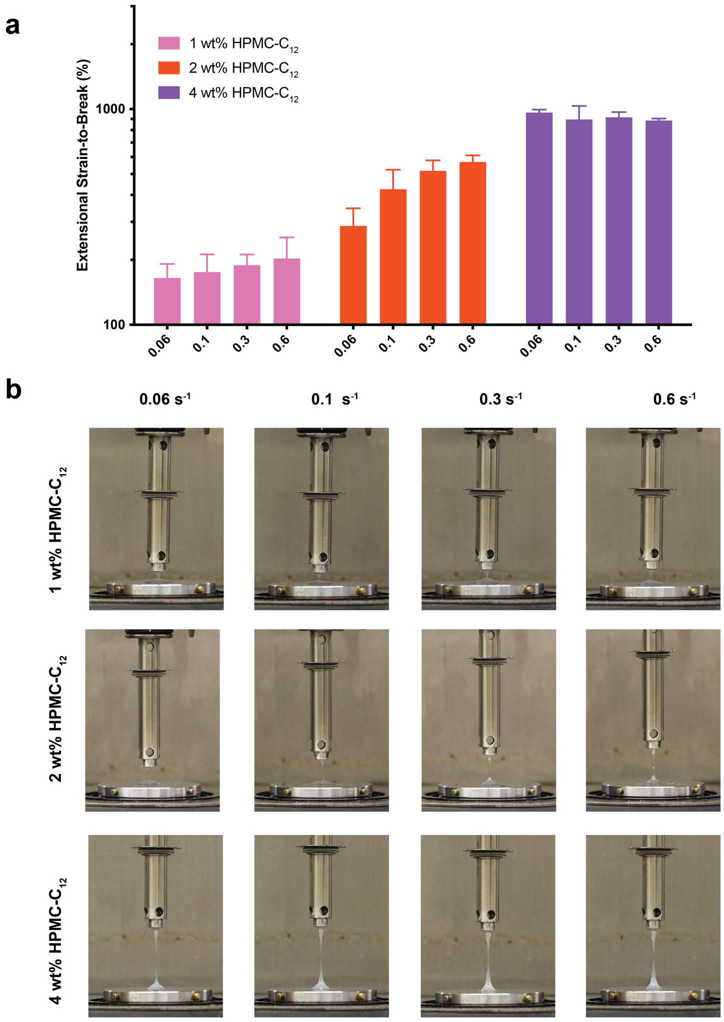

Supplementary Figure 11: Filament stretching extensional rheology of various alternative liquid polymer solutions of HPMC-C_{12}. **a,** Extensional strain-to-break measurements for all formulations. **b,** Representative images at of all formulations at the strain rates tested.

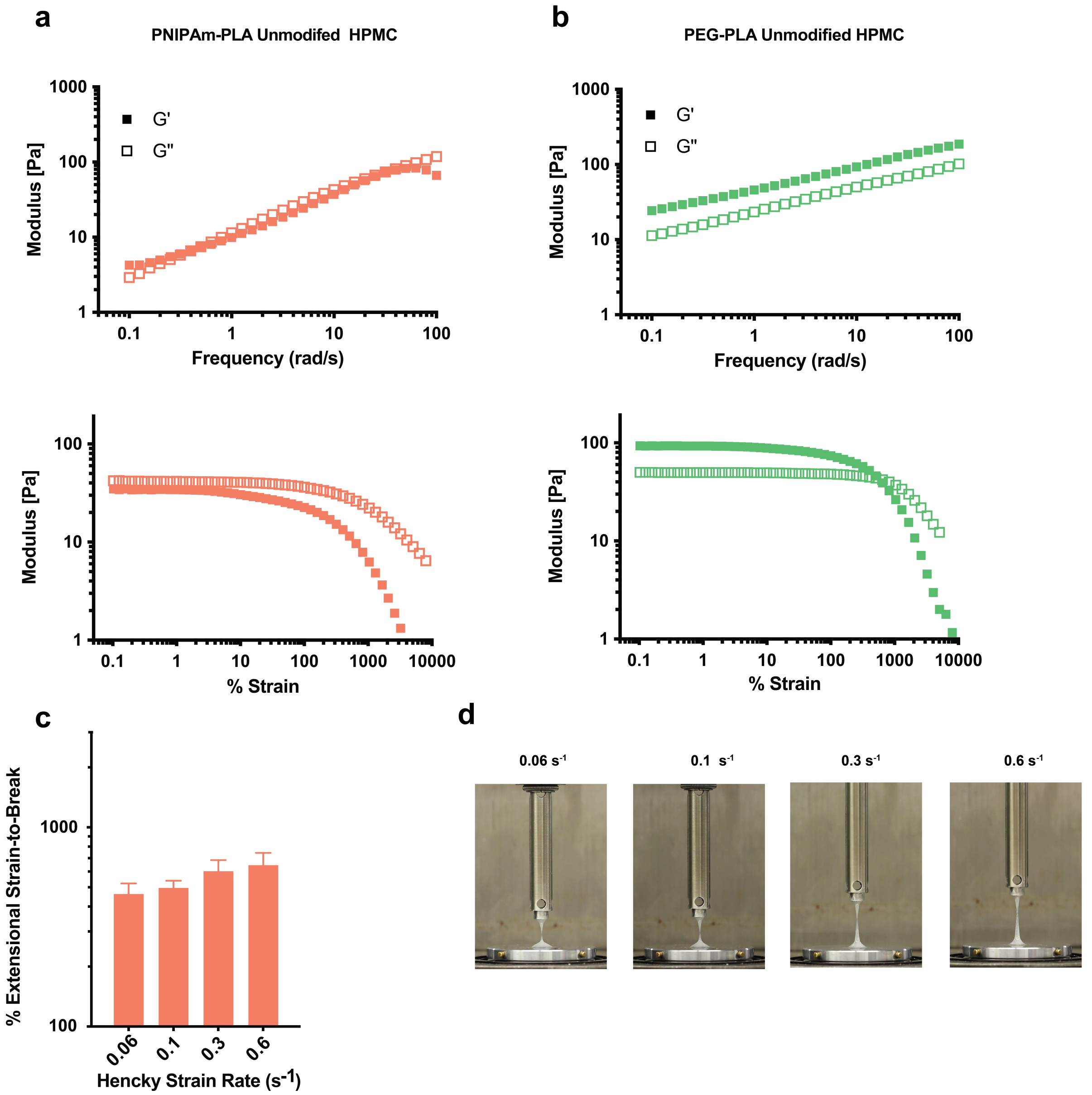

Supplementary Figure 12: **a,** Shear rheology (frequency sweep at 1% strain and amplitude sweep at 10 rad/s) of a PNP hydrogel with (50%) PNIPAm-PLA nanoparticles with unmodified HPMC (no dodecyl modification). **b,** Shear rheology (frequency sweep at 1% strain and amplitude sweep at 10 rad/s) of a PNP hydrogel with PEG-PLA nanoparticles with unmodified HPMC (no dodecyl modification). **c,** Filament stretching extensional rheology strain-to-break measurements of a PNP hydrogel with (50%) PNIPAm-PLA nanoparticles formulated with unmodified HPMC (no dodecyl modification). **d,** Representative images of filament stretching rheology of a PNP hydrogel with (50%) PNIPAm-PLA nanoparticles formulated with unmodified HPMC (no dodecyl modification).

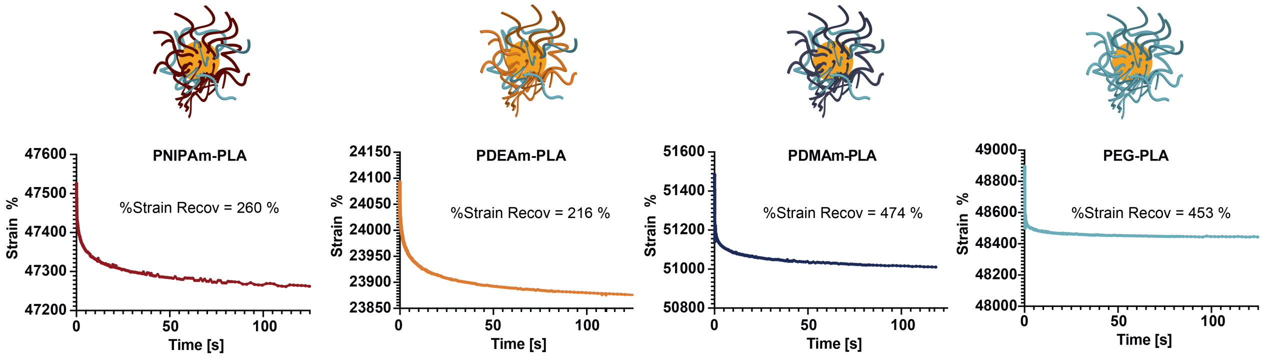

Supplementary Figure 13: Representative recoverable strain measurements for each PNP hydrogel formulation with the four groups of nanoparticles: (50%) PNIPAm-PLA, PDEAm-PLA, PDMAm-PLA and PEG-PLA.

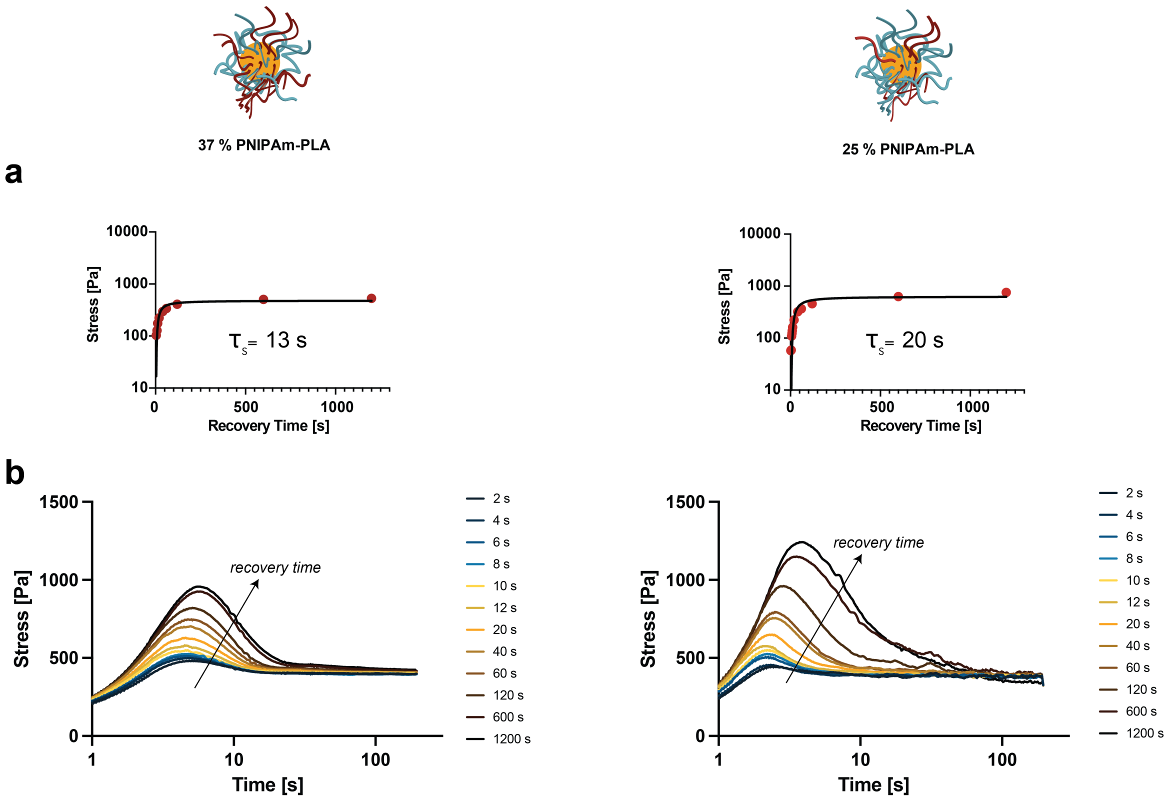

Supplementary Figure 14: a, Stress overshoot analysis with varying recovery times for PNP hydrogels formulated with nanoparticles containing 37% and 25% PNIPAm-PLA. Exponential fit to data shown in grey. Recovery times based on exponential fit denoted on corresponding graphs. **b,** Stress overshoot measurements with varying recovery times between imposed shear.

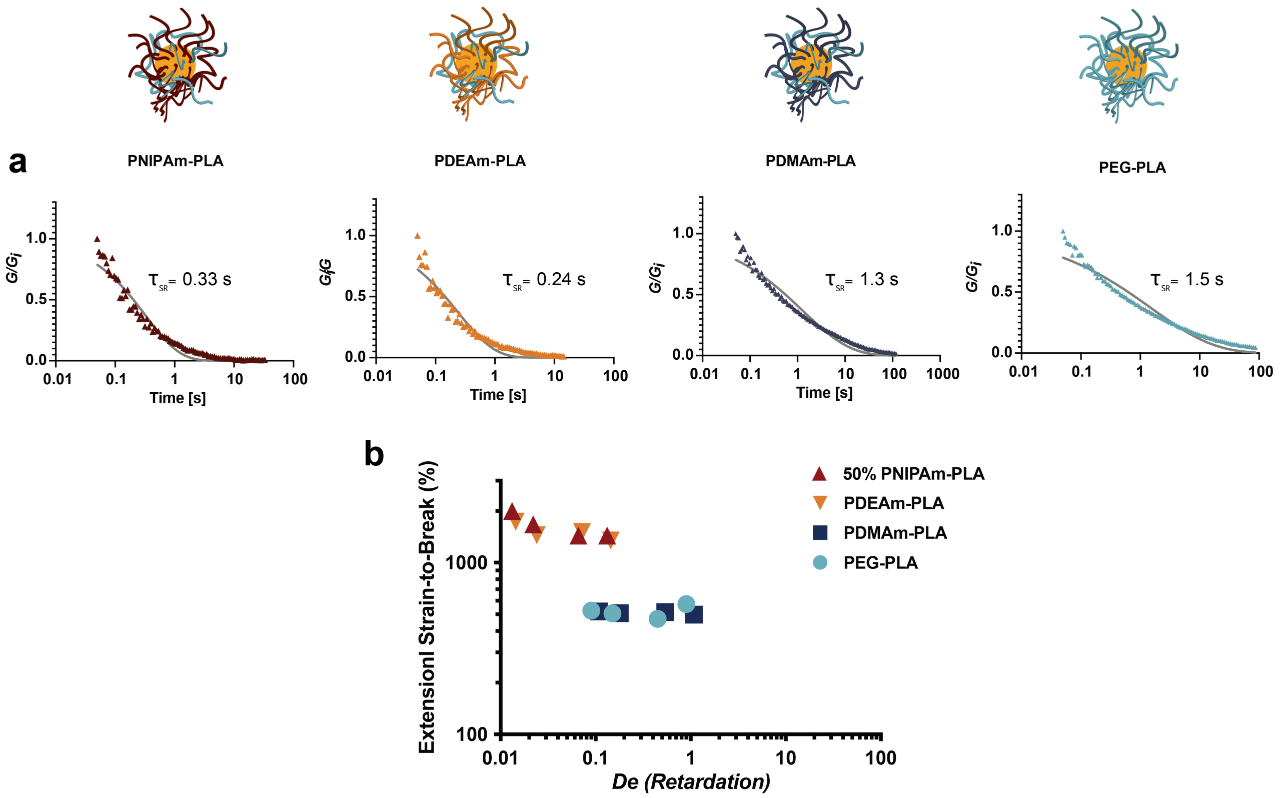

Supplementary Figure 15: **a,** Stress relaxation measurements of PNP hydrogels formulated with PNIPAm-PLA (50%), PDEAm-PLA, PDMAm-PLA and PEG-PLA. A normalized plateau modulus is plotted versus time. A fit to Kohlrausch's stretched-exponential relaxation model is shown in grey. The timescale extracted from this fit is shown on each corresponding graph. The corresponding values of the parameter a are 0.75, 0.71, 0.43, and 0.41. **b,** Dimensional analysis of Deborah number, De, based on the retardation timescale and strain rate timescales. A clear correlation is not apparent.

3 Supplemental Videos

Supplementary Video 1: Representative videos of filament stretching extensional rheology experiments of all formulations (PNIPAm-PLA, PDEAm-PLA, PDMAm-PLA, PEG-PLA, 37% PNIPAm-PLA, 25% PNIPAm-PLA) at 0.06 s^{-1}.

References

[1] Rauzan, B. M.; Nelson, A. Z.; Lehman, S. E.; Ewoldt, R. H.; Nuzzo, R. G. *Advanced Functional Materials* **2018**, *28*, 1707032.
